# Short-term effects of biochar amendment on root–microbe interactions in natural and peri-urban soils

**DOI:** 10.1038/s41598-026-46789-z

**Published:** 2026-04-09

**Authors:** Anna Gillini, Pamela Monaco, Gabriella Sferra, Antonio Bucci, Gabriella Stefania Scippa, Gino Naclerio, Dalila Trupiano

**Affiliations:** Laboratory of Urban Biodiversity, Department of Biosciences and Territory, University of Molise, 86090 Pesche, Italy

**Keywords:** Enzymatic activity, Hotspots, Root-microbe interactions, Microbial networks, Oak, Rhizomicrobiota, Ecology, Ecology, Environmental sciences, Microbiology, Plant sciences

## Abstract

Peri-urban areas are increasingly exposed to multiple environmental and anthropogenic threats that alter root-microbe interactions and compromise overall ecosystem stability. The short-term effect of biochar amendment on rhizoplane and rhizosphere interactions was investigated as an effective mitigation strategy, through rhizobox experiments. The higher pH, nutrient content, and metal(loid) concentrations in peri-urban soils compared to non-urban soils, lead roots to invest more in diameter and structural robustness rather than in exploratory growth, alongside a complex diversity in microbial communities. Biochar markedly affected the rhizosphere interactions in both non-urban and peri-urban soils by strengthening correlations among microbial taxa, soil nutrients, and root traits, while reinforcing pre-existing root development strategies. In the rhizoplane, biochar promoted new correlations between key microbial genera and soil/root features, likely creating microhabitats that support nitrogen cycling and plant growth-promoting interactions. Taken together, biochar appears to be an affordable mitigation strategy for improving root and microbial functional stability.

**Supplementary Information:**

The online version contains supplementary material available at 10.1038/s41598-026-46789-z.

## Introduction

The urban environment is a complex and dynamic system in which interactions between plant roots and the abiotic and biotic components of the soil play a crucial role^1,2^. Urbanisation can impact plant health and ecological functioning due to soil compaction, pollution, limited rooting area and fragmented green spaces^3–5^. In recent years, increasing urbanisation has led to the conversion of agricultural land and grasslands into urban sites, creating a transition zone, called peri-urban area, between the remaining rural sites and the urban areas^6^. Peri-urban areas are influenced by past land uses, such as agricultural activities and related human impacts, depending on local social and economic uses and practices^7^. Furthermore, urban expansion directly affects peri-urban areas, increasing landscape diversity, complexity, and fragmentation. These processes also intensify soil disturbances^8^, and create several challenges for root interactions^9^.

Some abiotic and biotic factors, such as the low soil permeability, extreme temperatures, and chemical contaminations, together with the presence of pathogens and/or invasive plant species, can interfere with natural soil-microbe-root interactions^9,10^, differently impacting plant roots and the microbial community composition and functioning^11,12^. In the context of potential interventions aimed at enhancing or restoring compromised ecosystems, soil amendments may provide valuable opportunities to improve soil fertility and microbial diversity. Several amendments have been shown to enhance key soil properties—including pH, structure, porosity, cation exchange capacity, water retention, and nutrient availability – demonstrating their potential suitability for peri-urban environments^13,14^.

An example of a sustainable management practice is the application of biochar^15,16^, a product obtained from the pyrolysis of biomass under oxygen-limited conditions^17,18^. Over the past decade, a considerable amount of research has demonstrated that biochar can improve soil physical properties, and increase or stabilize soil organic carbon pools^19^, reducing greenhouse gas emissions^20^. As a consequence, biochar has also been shown to enhance root development and growth in forest ecosystems, increasing the total root length, area and surface, and thereby favouring water and nutrient absorption. Furthermore, the biochar’s porous surface offers also stable conditions and an appropriate microhabitat for the soil microbial communities, and thus facilitating rhizosphere interactions toward increased resilience and functional efficiency^21–25^. Previous studies have shown that biochar amendments can shift microbial co-occurrence networks by increasing their complexity and connectivity, stimulate soil enzymatic activities involved in nutrient cycling, and enhance microbial diversity^26,27^. Most studies on biochar application have focused on changes in agricultural soil properties and the associated ecological processes^28–32^. However, in forest soils, biochar application generally enhances soil physio-chemical characteristics, improving plant growth and reshaping the microbial community structure, and increases soil microbial biomass^33^. All the aforementioned effects are highly biochar-, soil-, and plant-specific, and several key aspects have yet to be fully resolved. These include the examination on how biochar application can influence the diversity and complexity of the microbial communities characterizing two different compartments like the rhizoplane and the rhizosphere. The rhizosphere represents the soil volume surrounding the root system and that is directly influenced/modified by the plant activity, whereas the rhizoplane is composed by the root surface and its directly adhering soil and microorganisms which form the inner boundary of the rhizosphere^34,35^.

These two compartments are crucial for soil ecosystem functioning^36^ because resource inputs, such as organic matter and root exudates, are highly localized. This leads to the formation of microsites, known as “hotspots”, where plants, soil, and microorganisms interact^34,37^. Such hotspots are particularly sensitive to external disturbances, making their dynamics highly informative, especially in stressed or altered environments like urban and peri-urban soils^37,38^. Conventional methodologies provide limited insights into the vast array of rhizosphere and rhizoplane interactions^39,40^; therefore, little is known about these processes in disturbed environments. In this regard, the primary objectives of the present study were to apply an innovative set of integrated methodologies to investigate the potential effect of the biochar application on the interactions between *Quercus cerris* L. roots and the abiotic and biotic components in non-urban and peri-urban soils. This species was selected because it naturally occurs within the urban territory of Campobasso, where the soils for the experiments were collected, making it a relevant species for future applications in local urban and peri-urban areas.

Specifically, the short-term effects of biochar amendment on rhizoplane and rhizosphere interactions were investigated in a rhizobox experiment. Changes in soil parameters, root morphology, root exudates (in terms of extracellular enzymatic activity), and microbial community structure were analysed. Additionally, a network-based approach, supported by bioinformatics, was applied to analyse the microbial rhizosphere and rhizoplane communities’ structures and associations with root traits and soil characteristics. This approach provides a comprehensive understanding of the structural and functional properties of rhizosphere and rhizoplane ecosystems, while also revealing how biochar reshapes the belowground interactions and ultimately influences holobiont functionality across diverse environments.

Based on previously reported findings, we hypothesized that biochar application could modify rhizosphere and rhizoplane microbial communities and their interaction networks, potentially influencing soil characteristics, root morphological traits and enzyme activities.

## Materials and methods

### Soil sampling and characteristics

Soils samples from a natural non-urban (Bosco Faiete, BF; 41.55386986394396, 14.61640697891846) and a peri-urban (San Giovanni, SG; 41.58205965765106, 14.691131315095594) forest sites, located in Campobasso (Italy), were collected in April 2024—after receiving the related permits from the Municipality or private owners in the case of public or non-public spaces, respectively—and used for the rhizobox experiment. More specifically, the natural non-urban site is a part of the Special Area of Conservation “Monte Vairano” (IT7222295; Habitat Directive 92/43 EEC). The peri-urban site, instead, is characterised by the presence of a residual oak forest, which over time has been reduced and compressed by human infrastructures (roads, buildings and constructions) and agricultural activities, mainly focused on cereal cultivation. Both the sites are classified according to the Ecoregions of Italy as 1C3a1 (1 = Temperate Division, C = Apennine Province, 3 = Southern Apennine Section, a = Campanian Apennine Subsection; 1 = land unit) with the natural non-urban forest site principally composed of sandstones and conglomerates and the peri-urban site is characterized by feldspathic quartz sandstones^41^.

### Rhizobox set up: plants growth and treatment

*Quercus cerris* L. seeds were obtained from the plant nursery “Verde Idea” (Isernia – Italy – owner by Emiliano Neri) and germinated in a growth chamber as follows: the seeds were placed in rectangular trays (10 cm × 3 cm × 3 cm) filled with perlite (5 mm) and watered every 2–3 days under a photoperiod of 16 h of light and 8 h of darkness at 20 °C and 45% humidity. After molecular verification of the species following the protocol of Fantozzi et al.^42^ and the specimen assignment to *Q. cerris*, plants of 4 months, with root systems in active primary growth, were transferred to the rhizoboxes. For all plant materials no voucher specimen has been deposited in publicly available herbariums.

The rhizoboxes were filled with four different substrates: a natural non-urban forest soil (non-urban), a natural non-urban forest soil amended with biochar (non-urban + Biochar), a peri-urban forest soil (peri-urban) and the peri-urban forest soil with biochar (peri-urban + Biochar). Three plants were used for each substrate (3 rhizoboxes for each experimental group, Figure S1).

A 5-mm thick styrofoam panel wrapped in a transparent film was placed inside each rhizobox (dimensions: 20 cm × 15 cm × 3 cm, Tomix Design S.r.l.). Then, 300 g of the selected plant-growing substrates were sieved (1 mm texture) directly onto the film surface and moistened to 60% of their water holding capacity (WHC). The WHC of the substrates was determined at the beginning of the experiment to calculate the amount of water required to reach 60% of WHC for the soils used in each rhizobox. During the experiment, each rhizobox was periodically weighed, and water was added to restore the weight corresponding to 60% of WHC, ensuring that substrate moisture was maintained at a consistent level during the experiment.

In the biochar-amended treatments, 7 g of biochar were applied to the surface of 300 g of soil (the amount required to fill the rhizobox). This corresponds to an application rate of approximately 2.5% (w/w), a level documented to induce measurable changes in root growth and rhizosphere microbial communities. Surface application was chosen over homogeneous mixing to better simulate realistic field conditions^43–45^. Biochar was produced by the Romagna Carbone company (Italy) using biomass derived from orchard pruning through a slow pyrolysis process that takes an average of three hours to complete at 500°C^46^ (Table S1). Substrates physio-chemical characteristics were analysed by Eurofins Environ-lab s.r.l. (Corteolona and Genzone, Pavia) using the standard procedures described in the next paragraph (Table S1).

After substrate preparation, the oak plants were carefully placed in the rhizoboxes and secured with a second styrofoam paper enveloped in transparent film. Finally, the plexiglass panel of each rhizobox was closed, fixed with an elastic band, and covered with aluminium foil. The rhizoboxes were then placed at an inclination of 45°, using a specific support, in a growth chamber for 2 weeks (16 h of light and 8 h of darkness at a temperature of 20 °C and 45% humidity) (Figure S1).

At the end of the experiment, 2D zymography was performed, and roots and soil substrates collected for morphological, microbiological and chemical characterization.

### Soil substrate chemical analyses

Soil substrates, together with biochar samples, were sent to Eurofins Environ-lab s.r.l. (Corteolona and Genzone, Pavia) for their characterization, by using standard procedures (each measurement in triplicate). Specifically, the pH of the soil was measured with a pH meter according to ISO 10390:2021 guidelines. Nitrogen concentration was measured using the Kjeldahl method for total nitrogen (TN) and Devarda’s alloy for ammoniacal nitrogen (NH₃/NH₄⁺). Iron sulphate titration followed by dichromate oxidation was performed to assess the total organic carbon (TOC). The acid extraction followed by inductively coupled plasma optical emission spectrometry (ICP-OES; Perkin Elmer Optima 8000DV, Thermo Scientific, Germany) allowed to determine the concentration of total phosphorus (TP) and available phosphorus (Pav), the essential macronutrients [sodium (Na), boron (B), potassium (K)], and the potentially toxic elements, including both heavy metals and trace metals: arsenic (As), copper (Cu), lead (Pb) , nickel (Ni), cobalt (Co), zinc (Zn). The acid digestion of soil samples was performed using aqua regia, a mixture of concentrated hydrochloric acid (HCl) and nitric acid (HNO₃) in a 3:1 ratio, according to the procedure described in UNI EN ISO 54321:2021. In brief, a known amount of oven-dried soil was placed in a digestion vessel, and the aqua regia mixture was added. The sample was then heated under controlled conditions to facilitate the extraction of the elements of interest from the soil matrix. After cooling, the digestate was filtered and brought to volume with ultrapure water for subsequent elemental analysis.

The statistical analyses were conducted using R (version 4.2.2) for the ANOVA and Tukey post hoc considering three replicates for each treatment.

### Root morphological analysis

RhizoVision Explorer’s (Version 2.0.3), in "*broken root*" mode, was used to conduct root morphological analyses. Briefly, roots were meticulously removed from the substrate, cleaned with distilled water, separated and placed on a scanner surface with a ruler for reference. The resulting images were uploaded into RhizoVision Explorer to measure key root traits: the number of root tips, total root length (mm), network area (mm^2^), mean diameter (mm), median diameter (mm), maximum diameter (mm), perimeter (mm), volume (mm^3^), and surface area (mm^2^). Specific root length (SRL) was calculated by dividing the total root length by the total root dry mass calculated as dry weight (DW, mg) following two days of oven-drying at 80 °C.

For data visualization and analysis, R version 4.2.2 (R Core Team, 2021) was used, including the packages “tidyverse”^47^ and “rstatix”^48^.

### The 2D zymography

The 2D zymography approach was used to evaluate the spatial localization of the activity of three hydrolytic enzymes: β-glucosidase (β-Glu), leucine aminopeptidase (LAP), and acid phosphatase (Phos), involved in the C-, N-, and P-cycles, respectively. Each enzyme’s fluorogenic substrate [4-methylumbelliferyl (MUF)-β-D-glucopyranoside for β-Glu, L-leucine-7-amino-4-methylcoumarin (AMC) for LAP, and 4-MUF-phosphate for Phos] was dissolved in 300 μl of dimethyl sulfoxide (DMSO) and then diluted with a corresponding buffer solution: 0.1 M MES buffer for β-Glu and Phos, and 0.05 M TRIZMA buffer for LAP. The solutions were used to saturate a polyamide membrane (0.45 μm pore size) which was carefully placed over the exposed roots in the rhizoboxes. A plastic film was applied to prevent the membrane from drying, and a weight was placed on top to ensure contact with the roots. After an hour of incubation, the membrane was removed and exposed to UV light from two lamps (VILBER,VL.215 L.C.). A Canon camera was used to take the membrane images (ISO 800, shutter speed 1/8, aperture f/4), and the rhizobox photos (ISO 100, shutter speed 1/15, aperture f/4). This final step is essential to match the displayed zymograms with the plant roots. The calibration of each enzymatic activity was carried out as stated in Razavi et al.^49^.

Hotspot areas were analysed using the open-source software ImageJ^50^ and R (version 4.2.2), following the method outlined by Bilyera et al.^51^. Hotspots were defined as pixels with grey values greater than the "Mean + 2SD" threshold, derived from the previous grayscale histogram components. Considering the objectives of the study, different hotspot areas were calculated: the hotspot of the entire rhizobox area, the hotspots of the rhizospheric soil and the hotspots of the rhizoplane. The rhizoplane area was determined by overlapping the root’s images with the obtained zymograms and selecting the exact point of the root’s footprint on the zymogram.

Instead, the rhizosphere was considered as the region starting from the end of the root (influenced by the root’s activity) and the beginning of the bulk soil^52^. The hotspot area was determined in ImageJ as percentage based on the number of pixels in the hotspot and the number of pixels in the background using the following formula (formula 1):

1 $$\%Hotspot area= \frac{100\times (Number of pixels oin the hotspot)}{(Number of pixels in the hotspot-Number of pixels in the background)}$$

### Characterization of rhizosphere and rhizoplane microbial communities: DNA extraction, 16S rDNA amplicon sequencing, and bioinformatics analyses

For the microbiological investigations, three rhizospheric soil samples were collected with a heat-sterilized stainless-steel spoon from the three rhizoboxes of each experimental group (nine rhizospheric samples for each condition). Additionally, five root segments (approximately 2 cm long and less than 1 mm thick) were excised from each root system and pooled into a sterile tube using sterile scissors and forceps (three rhizoplane samples for each experimental group).

The rhizosphere and rhizoplane prokaryotic communities of *Q. cerris* L. seedlings were characterized through a molecular approach. In detail, the total genomic DNA was extracted from the rhizospheric soil samples using the DNeasy PowerSoil Pro Kit (Qiagen, Hilden, Germany), according to the manufacturer’s instructions. The rhizoplane communities were analysed following the protocol described by Pietrangelo et al.^53^ with some modifications. Briefly, the root segments were washed by shaking three times in 10 mL of sterile distilled water and once in 20 mL of sterile 0.85% NaCl solution. Roots were then subjected to a two-step sonication treatment using an ultrasonic processor (VCX130; Sonics & Materials, Inc., Newtown, USA) at a 20 kHz and 30% amplitude. The first sonication step, aimed at removing loosely attached microbial cells from the root surface, involved 2 min 30 s in 10 mL of sterile sonication buffer (0.85% NaCl, 0.1% Tween 80). The second sonication step, which enabled the detachment and recovery of the rhizoplane microorganisms, was performed in 10 mL of fresh sterile sonication buffer for 5 min. The obtained cell suspension was then adjusted to a final volume of 50 mL with sterile Milli-Q water and filtered through mixed cellulose ester membranes (47 mm diameter, 0.22 µm pore size; S-Pak, Millipore Corporation, Billerica, USA). Lastly, genomic DNA was extracted from the filters using the DNeasy PowerWater Kit (Qiagen, Hilden, Germany), following the manufacturer’s instructions.

Sequencing analyses were performed at BMR Genomics (Padova, Italy). Specifically, the V3–V4 regions of the 16S rDNA gene were amplified in a 35-cycle PCR with the primer pair Pro341F (5′-CCTACGGGNBGCASCAG-3′) and Pro805R (5′-GACTACNVGGGTATCTAATCC-3′), modified with universal tails^54^. Amplification products were purified using Thermolabile Exonuclease I (New England Biolabs), diluted 1:2, and further amplified in a second PCR with Nextera XT Indexes. Amplicons were normalized with SequalPrep (Thermo Fisher) and multiplexed. The pool was purified using Agencourt XP 1X magnetic beads before the library was run on the Illumina MiSeq and sequenced with V3 chemistry – 300PE strategy^12,55^.

Raw fastq files were processed with QIIME2 tools (version 2023.7)^56,57^. The reads were cleaned of primers employing the Cutadapt software (v. 2023.7) and processed with the denoised-paired plugin of the DADA2 software^58^. Briefly, sequences were trimmed at the 3′ end, filtered by quality and length, dereplicated, and merged to obtain unique sequences. As a final step, chimeras were removed. The resulting Amplicon Sequence Variants (ASVs) were associated with their abundance across the samples. The ASVs shorter than 370 bp were excluded to retain high-quality sequences consistent with expected amplicon size (460–470 bp) and remove truncated of potentially non-specific amplicons. Additionally, we applied a 0.005% relative abundance threshold to remove extremely rare ASVs likely resulting from sequencing artefacts, following commonly adopted microbiome filtering procedures^59^.

All reads were classified to the lowest possible taxonomic rank using a reference dataset from the SILVA database (version 138). Sequencing data generated in the present study have been deposited in the NCBI Sequence Read Archive (SRA) and are available under the accession number PRJNA1368845.

Alpha-diversity was calculated using the Shannon index, and differences between experimental conditions were tested with the Kruskal–Wallis test (*p* < *0.01*). Beta-diversity analyses were performed using the Bray–Curtis metric, and statistical comparisons were conducted using the PERMANOVA test (*p-value* < 0.01). Differential abundance analysis was carried out using R (version 4.2.2) and the packages “*dplyr*”^60^ and “*stats”*^61^*.*

### Network generation and analysis

Starting from the revealed abundance of the microbial genera, co-occurrence patterns were calculated to structure the microbiota using the function *rcorr* from the R package “Hmisc”^62^. Networks were constructed separately for each soil type (non-urban and peri-urban), compartment (rhizosphere and rhizoplane) and treatment (with and without biochar) selecting only highly significant correlations (*p* < *0.01*) between genera abundances. Both node-level centrality measures (degree and betweenness centrality), which help identify the roles of individual genera, and network-level topological measures—including modularity, average path length, clustering coefficient, and the number of nodes and edges—were considered to compare the networks^63^. Visualization and extraction of network metrics were performed in Cytoscape^64,65^.

To verify that the obtained networks reflected real ecological patterns rather than random structures, corresponding random networks (null models) were generated while maintaining the same number of nodes and edges. The topological metrics of the observed and random networks were compared, and networks with values significantly different from the null models (*p* < *0.05*) were considered non-random^66^.

Finally, a heat map was created to investigate associations among the microbial network modules (only the top four modules for each network), soil chemical parameters, and root traits. This analysis was conducted using the following packages: *ggplot2* (for graphical visualization,^67^), *dplyr*^60^ and *reshape2* (for data management and reshaping^68^), *vegan* (for ecological distance calculations and Mantel testing^69^), and *igraph* (for network creation and module recognition^70^). Spearman’s correlation analysis was applied to detect both positive and negative associations between microbial modules and soil or plant features. The only relationships accepted as significant were those with *p* < *0.01*. Mantel tests were also used to assess the overall correlation between environmental features and microbial modular organization.

## Results

### Soil substrate characteristics and root morphological traits

The analysis of soil properties (Table 1) revealed some significant differences between the non-urban and peri-urban soils. More specifically, soil pH, TOC, total and ammoniacal nitrogen, K, As, Ca, Co, Cu, Pb, Ni, Zn, and Mn were higher in peri-urban (p < 0.01) than in non-urban soil (Table 1).

**Table 1:** Chemical properties of soil substrates.

Soil chemical properties	Non-urban	Peri-urban	Non-urban + Biochar	Peri-urban + Biochar
pH	6.5 ± 0.13 a	7.6 ± 0.17 b	5.9 ± 0.06 a	7.6 ± 0.05 b
TOC (%dm)	1.3 ± 0.05 a	2.3 ± 0.2 b	1.5 ± 0.01 a	2.3 ± 0.05 b
TN (%dm)	0.09 ± 0.01 a	0.2 ± 0.03 b	0.11 ± 0.1 a	0.2 ± 0.02 b
N_NH3-NH4_ (mg/Kg dm)	45.9 ± 4.1 a	12.1 ± 0.9 b	42.7 ± 1.3 a	13.1 ± 1.9 b
NN (%dm)	0.76 ± 0.03 a	1.01 ± 0.31a	7.8 ± 0.2 b	6.06 ± 0.4 c
TP (% dm)	0.01 ± 0.01 a	0.01 ± 0.01 a	0.01 ± 0.02 a	0.041 ± 0.02 a
P_av_ (mg/Kg dm)	< 1 a	< 1 a	< 1 a	< 1 a
B (mg/Kg dm)	< 5 a	< 5 a	< 5 a	< 5 a
Na (mg/Kg dm)	102.6 ± 5.5 a	169.3 ± 54.6 a	100 ± 0.2 a	173.3 ± 34.1 a
K (mg/Kg dm)	593. 3 ± 192.9 a	2933 ± 40.6 b	643.3 ± 120.9 a	2888 ± 933 b
As (mg/Kg dm)	1.9 ± 0.05 a	4.3 ± 0.4 b	1.9 ± 0.01 a	4 ± 0.8 b
Ca (mg/Kg dm)	1186 ± 122.4 a	7526.3 ± 142 b	1039.6 ± 78.7 a	8196 ± 1270 b
Cd (mg/Kg dm)	< 0.35 a	< 0.35 a	< 0.35 a	< 0.35 a
Cr (mg/Kg dm)	9.1 ± 0.1 a	26 ± 14 a	9.2 ± 0.2 a	22.6 ± 9.6 a
Co (mg/Kg dm)	2.92 ± 0.2 a	10.4 ± 1.6 b	3.6 ± 0.07 a	2.11 ± 9.5 b
Cu (mg/Kg dm)	3.8 ± 0.5 a	19.6 ± 2.8 b	4.8 ± 0.2 a	18.7 ± 3.6 b
Pb (mg/Kg dm)	7.7 ± 0.7 a	15.1 ± 1.7 b	8.9 ± 0.7 a	13.8 ± 2.8 b
Hg (mg/Kg dm)	< 0.3 a	< 0.3 a	< 0.3 a	< 0.3 a
Ni (mg/Kg dm)	4.8 ± 0.9 a	25.4 ± 5.5 b	6 ± 0.5 a	23.5 ± 5.1 b
Zn (mg/Kg dm)	19 ± 3 a	57 ± 13.7 b	20.3 ± 0.5 a	53 ± 12.2 b
Mn (mg/Kg dm)	453 ± 46.7 a	814 ± 127.8 b	519.3 ± 37.6 ab	798.6 ± 178 b

Main soil chemical parameters measured in the different soil substrates [non-urban; non-urban + biochar (B); peri-urban; peri-urban + B]: TOC—Total Organic Carbon, TN—Total nitrogen, N_NH3-NH4—_ammoniacal nitrogen_,_ NN—nitric nitrogen, TP—Total phosphorus, P_av_—available phosphorus, B—Boron, Na -Sodium, K—Potassium, As—Arsenic, Ca—Calcium, Cd—Cadmium, Cr—Chromium, Co—Cobalt, Cu—Copper, Pb—Lead, Hg—Mercury, Ni—Nickel, Zn – Zinc and Mn—Manganese. The values are shown as a mean of three replicates ± standard deviation and are expressed as a percentage of dry matter (dm) or mg per Kg^-1^ (mg/Kg) of dry matter. The statistical differences were tested with One-way ANOVA and Tukey’s post-hoc test at a significance level of *p* < *0.05*. Values with different letters indicate statistical differences among soil substrates (*p* < *0.05*), whereas the same letter indicates no significant differences.

Considering the effect of biochar amendment, only nitric nitrogen amount was increased in both non-urban (*p* = *2.58e-06*) and peri-urban (*p* = *1.20e-04*) soils.

The morphological root parameters showed significant variation across soil substrates. Specifically, non-urban soil revealed significantly higher values of total root length (*p* = *0.006*) and network area (*p* = *0.005*) compared to peri-urban soil. On the contrary, the peri-urban soil showed significantly highest values of branching frequency (*p* = *0.03*), perimeter (*p* = *0.002*) and specific root length (*p* = *0.02*) (Table 2).

**Table 2:** Root morphological traits.

Root traits	Non-urban	Non-urban + Biochar	Peri-urban	Peri-urban + Biochar
Tips	3173.5 ± 329 a	4627.5 ± 424 b	3818.5 ± 315 ab	3792 ± 437ab
BP	3679 ± 376 a	5593.5 ± 304 b	4355.5 ± 348 a	3207 ± 724 a
TRL	6841.6 ± 482 a	6638.1 ± 150 a	4658.2 ± 390 b	6730.7 ± 458 a
BF	0.85 ± 0.02 a	0.85 ± 0.02 a	0.85 ± 0.04 b	0.85 ± 0.05 b
NetA	4040.6 ± 316 a	2724.1 ± 191 b	2550 ± 254 b	4341.1 ± 637 a
AD	1.8 ± 0.03 a	1.3 ± 0.2 b	1.9 ± 0.1 a	2.1 ± 0.2 a
MD	0.7 ± 0.06 a	0.6 ± 0.09 a	0.8 ± 0.01 a	1.1 ± 0.1 b
Max D	15.4 ± 0.8 a	12.1 ± 0.1 a	17.6 ± 1 a	26.2 ± 2 c
P	3228.5 ± 337 a	3983 ± 102 b	4753.2 ± 62 c	4540.5 ± 27 c
V	47,820.2 ± 14,403 a	21,468.5 ± 4258 a	25,377.3 ± 12,199 a	126,443.5 ± 94,843 a
SA	54,024.5 ± 7291 a	23,285.4 ± 576 b	23,394.3 ± 3012 ab	37,830.4 ± 13,832 ab
SRL	372.9 ± 17 a	516.2 ± 63 b	500.3 ± 52 b	305.7 ± 6 a

Main root morphological traits measured on *Q. cerris* plants grown on different soil substrates [non-urban; non-urban + biochar (B); peri-urban; peri-urban + B]: Tips – number of tips, BP—branching points, TRL—total root length (mm), BF—branching frequency, NetA -network area (mm^2^), AD—average diameter (mm), MD—median diameter (mm), Max D—maximum diameter (mm), P—perimeter (mm), V—volume (mm^3^), SA—surface area (mm^2^), SRL—specific root length (mm/mg). All the values are expressed as a mean of three replicates ± standard deviation. Values with different letters indicate statistical differences among soil substrates (*p* < *0.05*), whereas the same letter indicates no significant differences.

Most of the root morphological traits showed significant variations also related to biochar amendment (Table 2). More specifically, in the non-urban soil, biochar application decreased the network area (*p* = *0.01*), maximum diameter (*p* = *0.04*), average diameter (*p* = *0.02*) and surface area (*p* = *0.006*) while it increased specific root length (*p* = *0.01*), number of tips (*p* = *0.006)* and perimeter (*p* = *0.003*). In the peri-urban soil, only the specific root length (*p* = *0.003*) was decreased by biochar amendment while the network area (*p* = *0.002*), median diameter (*p* = *0.007*), maximum diameter (*p* = *0.0001*), and total root length (*p* = *0.003*) were increased (Table 2).

### 2D zymography

**Fig. 1:**
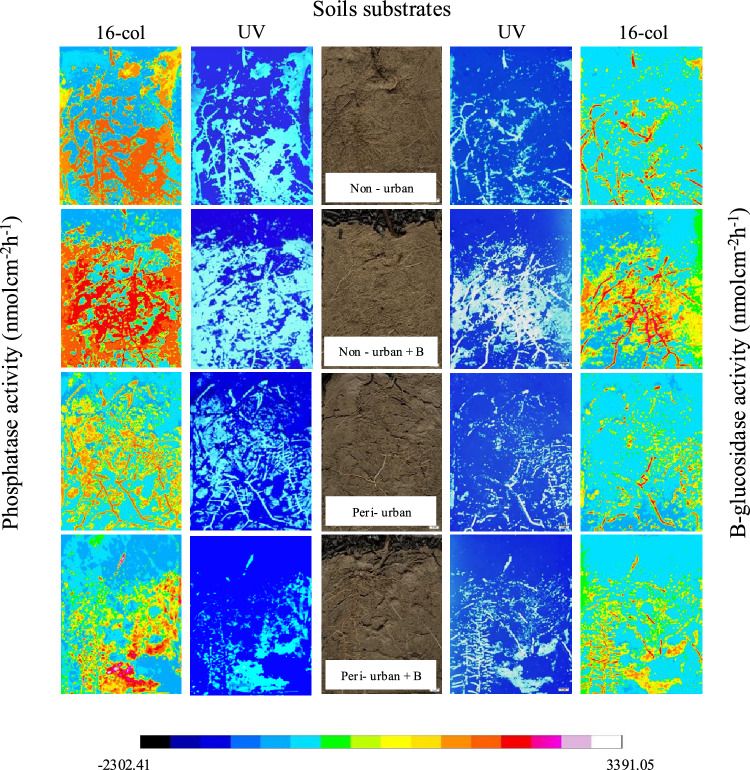
Soil zymography results considering the enzymatic activity of ß-glucosidase (ß-Glu) and acidic phosphatase (Phos) expressed as nmol cm^-2^ h^-1^. Central panel reports the images of the rhizoboxes growing *Q.cerris* plants on different soil substrates [non-urban soil; non-urban soil + biochar (B); peri-urban soil; peri urban soil + B], middle panles report the images of the hotspot area under UV light (UV) and lateral panel reports the images of the zymograms obtained using the 16 colors mode of ImageJ (16-col).

The soil zymography highlighted significant differences in the extracellular enzymatic activity among the soil substrates. In detail, the ß-Glu was higher in non-urban than in peri-urban soil (ß-Glu: non-urban = 548 ± 12.8 nmol cm-^2^ h-^1^; peri-urban = 231.7 ± 33.4 nmol cm-^2^ h-^1^; *p* < *0.01*) while both Pho (non-urban = 1882 ± 149 nmol cm-^2^ h-^1^; peri-urban = 1929 ± 227 nmol cm-^2^ h-^1^; *p* < *0.07*) and LAP showed similar values (non-urban = 212 ± 18 nmol cm-^2^ h-^1^; peri-urban = 220 ± 17 nmol cm-^2^ h-^1^; *p* < *0.1*). The enzymatic activity of ß-Glu was significant intensified in biochar amended non-urban (non-urban = 548 ± 12 nmol cm-^2^ h-^1^; non-urban + biochar = 611 ± 11 nmol cm-^2^ h-^1^; *p* = *0.02*) and peri-urban (peri-urban = 231 ± 33 nmol cm-^2^ h-^1^; peri-urban + biochar = 469 ± 17 nmol cm-^2^ h-^1^; *p* = *2.8e-05*) soils (Fig. 1 and S2), LAP was unaffected and, instead, Phos activity increased significantly only in non-urban biochar amended soil (non-urban = 1101 ± 149 nmol cm-^2^ h-^1^; non-urban + biochar = 1882 ± 279 nmol cm-^2^ h-^1^; *p* = *0.04*). Considering the extent of the hotspots, biochar increased the percentage of the hotspot area of the ß-Glu enzyme in both soils (non-urban = 37% ± 3.7; non-urban + biochar = 60.5% ± 10.3; peri-urban = 31.8 ± 7.8; peri-urban + biochar = 48.9 ± 5.8; *p* = *0.009*). In the case of phosphatases, biochar had no effect in peri-urban soil but increased both enzyme activity and the percentage of hotspots in non-urban soil (non-urban = 55.7% ± 5.1; non-urban + biochar = 68.5% ± 5.2; *p* = *0.01*) (Fig. S2 and S3). However, despite in all experimental groups the three enzymatic activities were significantly higher in the rhizoplanes than in the rhizospheres (Fig. S3), no significant differences were observed between untreated and biochar treated soils in the two compartments (rhizoplane and rhizosphere).

### Characterization of rhizosphere and rhizoplane microbial communities

A total of 1,027,082 final reads (ranging from 7,376 to 47,312 per sample) were obtained from NGS analysis, following quality filtering and processing of the raw sequencing data. All reads were assigned to the *Bacteria* domain, while no *Archaea* were detected.

**Fig. 2:**
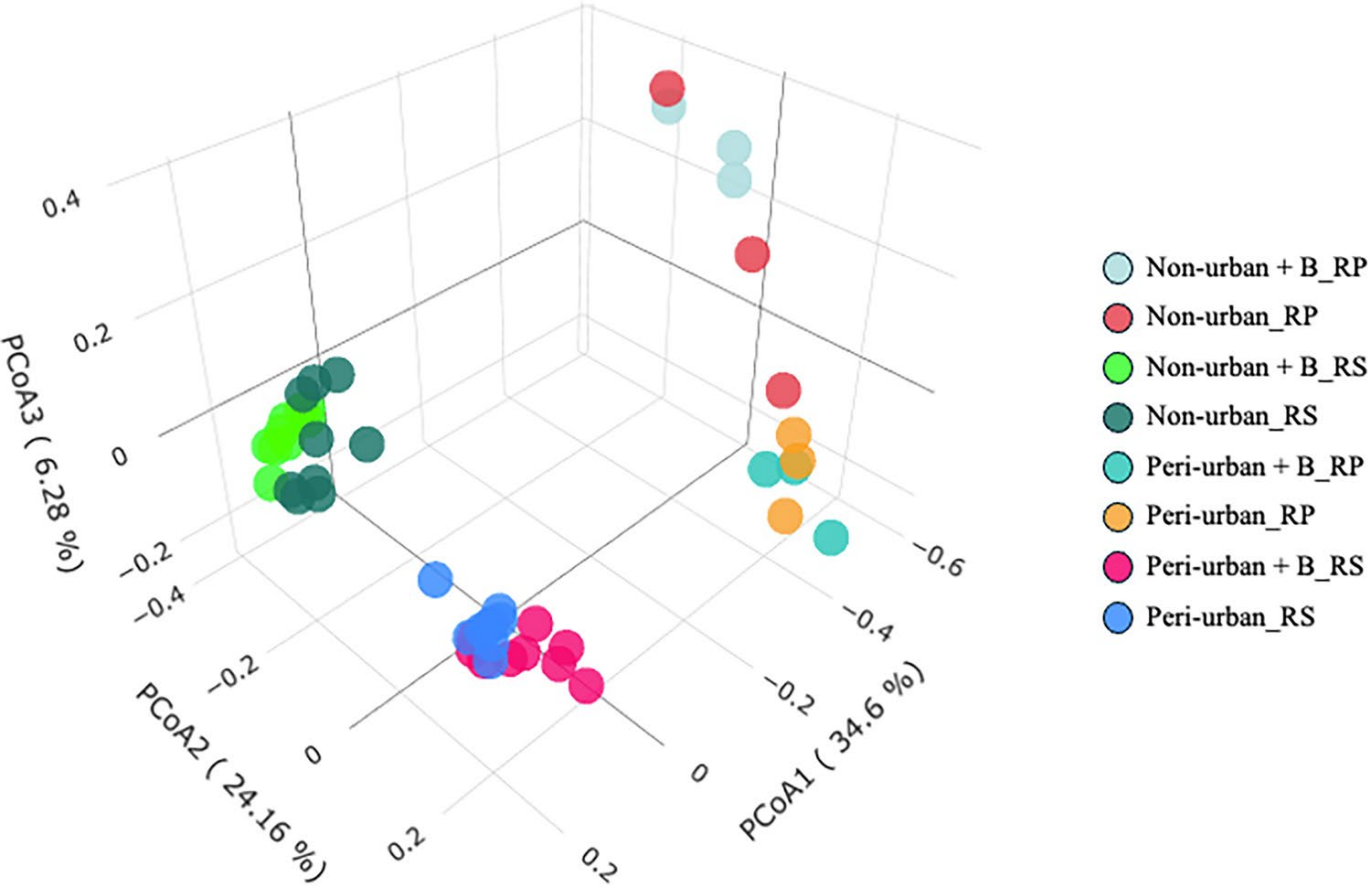
Principal Coordinate Analysis (PCoA) of microbial beta-diversity based on the Bray–Curtis metric. In the scatter plot, each sphere represents a microbial community, coloured according to the soil substrates (non-urban and peri-urban soils, untreated and biochar-treated) and compartments (rhizosphere/rhizoplane). B = biochar.

Considering all samples as a whole, no statistically significant differences were observed in terms of alpha-diversity between compartments (rhizosphere *vs* rhizoplane) or soil treatment (unamended *vs* amended), whereas considerable variations emerged depending on the provenance area of soil samples (non-urban *vs* peri-urban; *p-value* and *q-value* = 0.001), with peri-urban microbial communities exhibiting higher Shannon index values. Beta-diversity analyses, based on the Bray–Curtis metric, revealed a clear distinction between the rhizosphere and rhizoplane prokaryotic communities (*p-value* and *q-value* = 0.001), more pronounced within the peri-urban experimental group (*pseudo-F* = 21.39) compared to the non-urban (*pseudo-F* = 12.85). However, no statistically significant variations were observed in relation to biochar treatment (*p-value and q-value* = 0.210) (Fig. 2). Going to analyse differences in the two compartments (rhizoplane and rhizosphere) between the soil substrates, statistically significant differences emerged between peri-urban and non-urban microbial communities, both in the rhizoplane (*p-value* and *q-value* = 0.001; *pseudo-F* = 5.46) and in the rhizosphere (*p-value* and *q-value* = 0.001; *pseudo-F* = 47.84). Biochar treatment also induced significant changes in terms of beta-diversity in the rhizosphere microbial communities of both the peri-urban (*p-value* = 0.004*; q-value* = 0.009*; pseudo-F* = 2.38) and non-urban (*p-value* = 0.003*; q-value* = 0.009*; pseudo-F* = 4.51) soils. Moreover, in the rhizosphere soil samples (regardless of their provenance), biochar treatment resulted in a significant alpha-diversity increase (*p-value* = 0.006; *q-value* = 0.018), which was not detected within the rhizoplane communities.

**Fig. 3:**
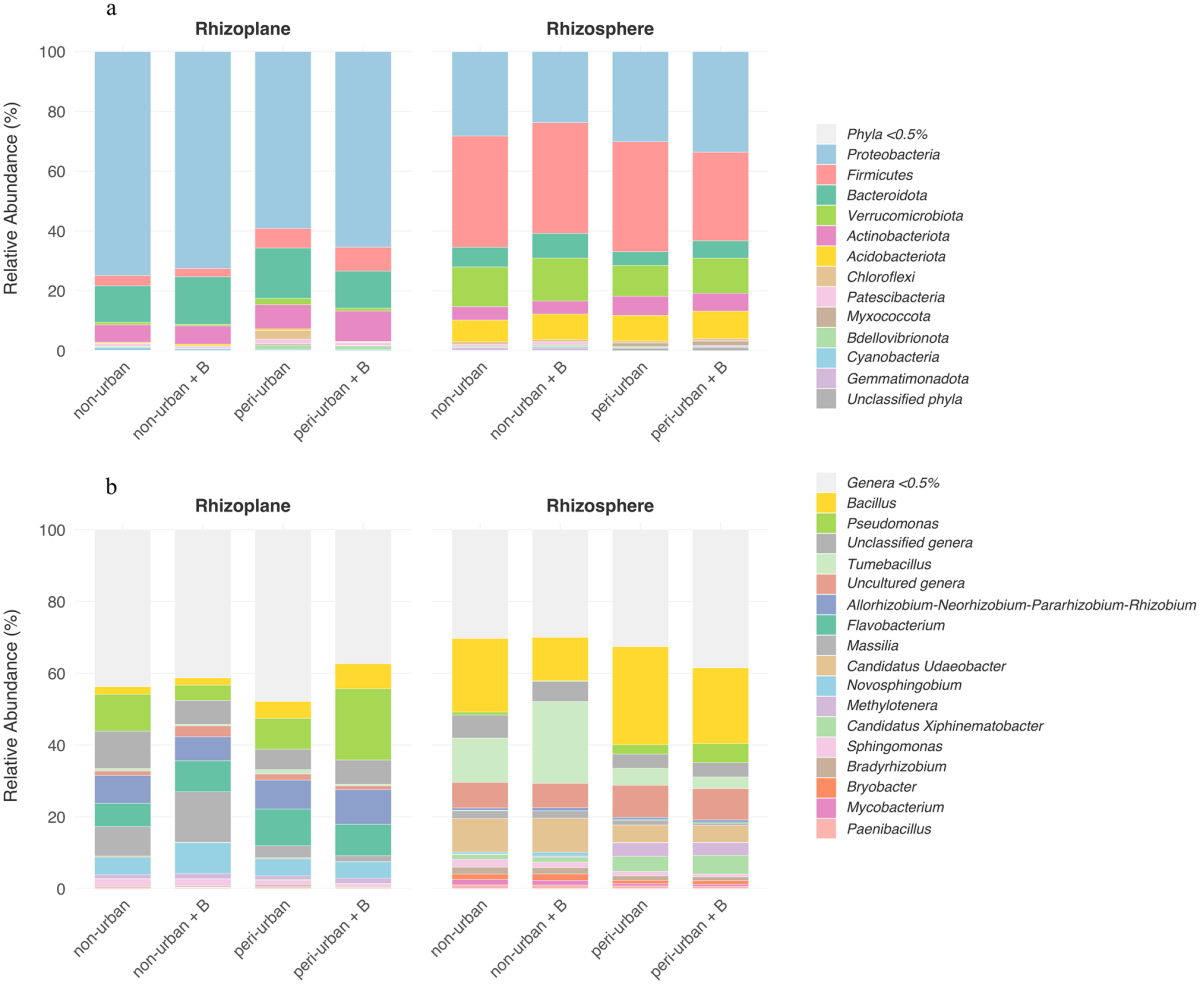
Rhizosphere and rhizoplane prokaryotic community composition at phylum and genus taxonomic level. (**a**) Taxonomic composition of the rhizosphere and rhizoplane prokaryotic communities at phylum level across the four experimental groups [non-urban, non-urban + biochar (B), peri-urban, peri-urban + B]. (**b**) Taxonomic composition of the rhizosphere and rhizoplane prokaryotic communities at genus level across the analysed experimental groups.

To outline the taxonomic composition of the microbial communities associated with *Q. cerris* roots across the four experimental groups, the mean relative abundance of each microbial taxon at the phylum and genus levels, was calculated from individual sample values between the compartments (rhizosphere and rhizoplane) and soil substrates. Important variations in the relative abundance of the main taxa between rhizosphere and rhizoplane communities already emerged at phylum level. In particular, *Acidobacteriota* (0.19–0.62%), *Firmicutes* (2.76–7.98%), and *Verrucomicrobiota* (0.51–2.07%) were less represented in the rhizoplane compared to the rhizosphere, where these phyla reached relative abundance values of 7.21–8.99%, 30.52–37.12%, and 10.32–14.31%, respectively (Fig. 3a; Table S2). Conversely, with percentages of 59.18–74.81%, 12.18–16.88%, and 5.81–10.19%, *Proteobacteria*, *Bacteroidota*, and *Actinobacteriota* were more abundant in the rhizoplane than in the rhizosphere (23.72–33.18%, 4.61–8.26%, and 4.38–6.42%, respectively). Although less represented, *Bdellovibrionota*, *Cyanobacteria*, *Gemmatimonadota*, *Myxococcota*, and *Patescibacteria* phyla contributed to determining the differences observed in the microbiota composition. Specifically, *Bdellovibrionota*, *Cyanobacteria*, and *Patescibacteria* were more abundant in the rhizoplane, whereas *Gemmatimonadota* and *Myxococcota* were enriched in the rhizosphere (Fig. 3a; Table S2). Beyond the differences observed between the two compartments, some bacterial phyla also showed variations in mean relative abundance values depending on soil type (non-urban *vs* peri-urban) and biochar treatment (Fig. 3a; Table S3). Indeed, differential abundance analysis at phylum level revealed that, in the rhizosphere, peri-urban soil showed the abundance of *Actinobacteriota* and *Myxococcota* to be significantly increased, whereas *Gemmatimonadota* and *Patescibacteria* decreased, compared to those of non-urban soil (Table S3). Moreover, *Armatimonadota* decreased and *Patescibacteria* increased in non-urban soil under biochar amendment (non-urban + B), while *Firmicutes* were depleted in biochar-treated peri-urban soil (peri-urban + B). Conversely, no significant variations in mean relative abundance percentages were detected in the rhizoplane communities between both non-urban and peri-urban soil and treated and untreated groups (Table S3).

As expected, a clear distinction in the taxonomic composition between rhizosphere and rhizoplane communities was also evident at the genus level (Fig. 3b; Table S4). Focusing on the most represented genera (i.e., those with mean relative abundances exceeding 0.5%), the prokaryotic communities of *Q. cerris* rhizosphere exhibited a greater heterogeneity, whereas the rhizoplane populations were dominated by a limited number of bacterial taxa: *Allorhizobium-Neorhizobium-Pararhizobium-Rhizobium* (6.70–9.69%), *Bacillus* (2.04–6.98%), *Flavobacterium* (6.48–10.24%), *Massilia* (1.65–14.04%), *Novosphingobium* (4.54–8.66%), and *Pseudomonas* (4.26–19.86%). Interestingly, apart from *Bacillus* (12.11–27.35% in the rhizosphere), these genera showed a notable increase in the rhizoplane compared to the rhizosphere, where they presented the following percentages: 0.54–0.84% (*Allorhizobium-Neorhizobium-Pararhizobium-Rhizobium*), 0.04–0.28% (*Flavobacterium*), 0.60–2.11% (*Massilia*), 0.07–1.08% (*Novosphingobium*), and 0.18–5.28% (*Pseudomonas*). Conversely, *Bradyrhizobium*, *Bryobacter*, *Candidatus_Udaeobacter*, *Candidatus_Xiphinematobacter*, and *Tumebacillus* prevailed within the rhizosphere communities, with relative abundance values of 1.05–1.93%, 0.92–1.78%, 4.70–9.49%, 1.34–5.26%, and 3.26–22.81%, respectively. In the rhizoplane, these latter bacterial genera did not exceed a mean relative abundance of 1.20% (observed for *Tumebacillus*), with *Bryobacter* showing the minimum percentage of 0.02% (Fig. 3b; Table S4).

Going on differential abundance analysis at the genus level, five microbial genera were differentially represented in the rhizoplane of *Q. cerris* roots grown in non-urban and peri-urban substrates: three genera (*Domibacillus*, *Chryseolinea*, and *Solibacillus*) were more abundant in peri-urban soils, whereas *C0119* and *Methylorosula* were more represented in non-urban substrates (Table S5).

In the rhizosphere, 84 differentially represented bacterial genera were found between non-urban and peri-urban soils. Moreover, in non-urban soils, biochar application resulted in the enrichment of *A21b*, *ADurb.Bin063-1*, *Inquilinus*, *LWQ8*, uncultured species of *Obscuribacteraceae*, *Pedosphaera*, and *Puia*, and in the depletion of *KD4-96*, *Microlunatus*, and *Pandoraea* (Table S5).

On the other hand, the biochar treated peri-urban soils were enriched in *BIrii41*, *Curvibacter*, *Gemmatimonas*, and *MND1* and depleted in *Paenisporosarcina*. However, with few exceptions, these bacterial genera exhibited mean relative abundance values below 0.5% across all investigated groups (Table S5).

### Microbial co-occurrence network analysis and hub genera

The co-occurrence networks were calculated to structure the microbiota communities in the different soil substrates and compartments. All parameters of the observed networks were statistically different respect those of randomized ones which assess them as good non-random models of the related communities (Table S6).

In detail, in the rhizosphere, the non-urban soil substrates network consisted of 173 nodes and 3013 edges, while in the biochar-treated samples the network was made of 168 nodes and 2333 edges. Instead, for peri-urban soil substrates, the rhizosphere network was characterized by 196 nodes and 3602 edges in untreated soils and 183 nodes and 3031 edges, in biochar treated soils.

When considering the rhizoplane, in the non-urban soil the network was made of 159 nodes and 6410 edges while the node and edges numbers diminished to 143 and 4165 respectively in the non-urban soil treated with biochar. In the peri-urban soil, the network was characterized by 167 nodes and 6258 edges and in the network of peri-urban soil treated with biochar the nodes are 163 and the edges are 5716.

Interestingly, the biochar revealed also effects on other network parameters (Table S6). Both in the rhizoplane and rhizosphere, the non-urban soil showed a network characterized by lower values of the degree centrality under biochar treatment respect the one of untreated soil, while in peri-urban soil network the degree centrality was significantly higher in biochar treated soil respect the untreated one. Differently, the betweenness centrality resulted not affected by biochar treatment (Figure S4).

**Fig. 4:**
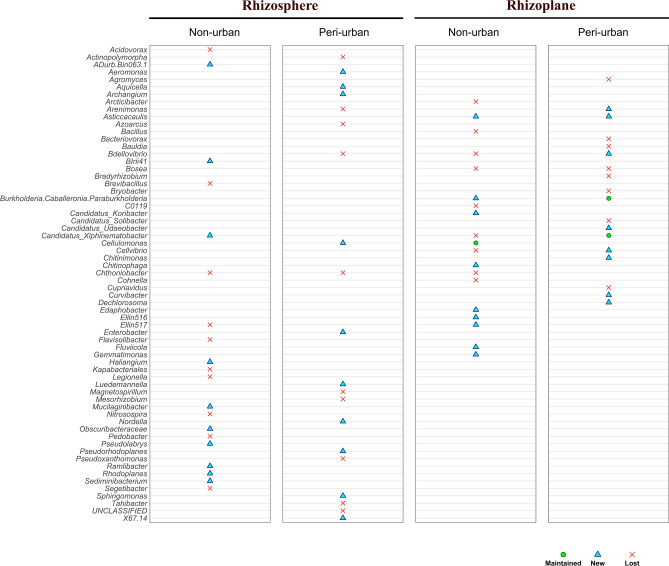
Shift of the hub microbial genera in rhizosphere and rhizoplane of soil substrates (non-urban and peri-urban soil) after biochar adding. The ‘x’ indicates microbial genera that were previously reported as hubs in the untreated soils and lost their hub role after biochar adding, the “triangle” indicates microbial genera that emerged as hubs under biochar treatment and that were not previously reported as hubs in the untreated soils, and the “circle” indicates genera that remained with hub roles in both the untreated and biochar treated soils.

Based on the degree, the top 10 microbial genera with crucial roles in the microbial community were selected for each network and deemed as network hubs. These were compared to observe the shift of genera assuming, maintaining or losing the role of hub under the biochar addition. Results showed that, in the rhizoplane and in the rhizosphere of both non-urban and peri-urban soil, the genera acting as hubs were strongly or completely restructured under biochar treatment (Fig. 4). Specifically, in the rhizosphere the biochar addition induced a shift of the totality of the hub genera of non-urban and peri-urban soil (Fig. 4, left panels).

In the rhizoplane, the biochar addition induced a massive shift of the hub genera in non-urban soil, with 9 out of 10 hubs being replaced and only one hub being maintained after amendment addition; in the peri-urban soil, instead, while 2 out of 10 genera were maintained, 8 out of 10 hubs underwent to shift after biochar addition (Fig. 4, right panels).

**Fig. 5:**
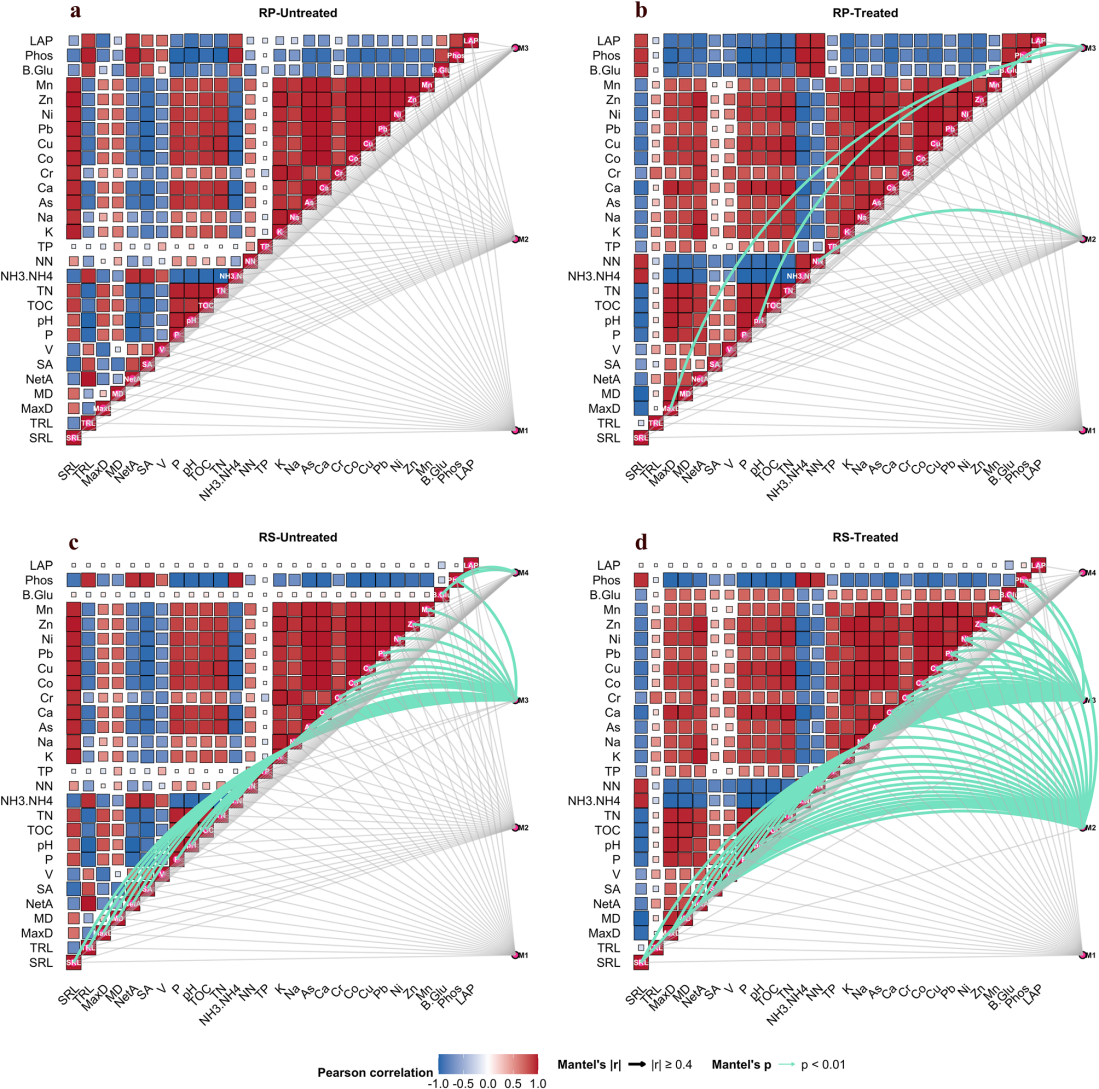
Heat map generated correlating the root traits, enzymes and the soil chemical parameters (*p* < *0.05*) with the associations (edges) with microbial genera clustered in the modules. The significant associations are shown as green edges (Mantel test *p* < *0.01and r* > *4*) while those not significant in grey. RS: rhizosphere; RP: rhizoplane; Untreated: non-urban and peri-urban soils; Treated: non-urban and peri-urban sols amended with biochar.

Additionally, a correlation-based approach was adopted to model and characterize the reciprocal dependencies occurring in the root-soil-microbiome systems of the two compartments under analysis. In detail, microbial genera were firstly clustered into modules according to their co-occurrence patterns in each network (Table S7). The successive correlation analysis showed significant associations among microbial modules, soil chemical parameters and/or root traits within rhizosphere and rhizoplane compartments modulated by biochar (Fig. 5). In the rhizoplane, no associations among microbial modules and soil or root parameters were found in unamended soil, while 4 new associations were induced as a consequence of biochar treatment (*p* < *0.05*, Fig. 5a and b; Table 3 and Table S8). On the other hand, in the rhizosphere, 24 significant associations normally occurred among the microbial community modules, the soil/root parameters and enzymatic activities; biochar amendment reinforced associations, with 22 maintained, 29 new and 2 missed associations (Fig. 5c and d; Table 3 and Table S8).

**Table 3:** Rhizoplane and rhizosphere associations between microbial community modules, the root/soil features and enzymatic activities modulated by biochar amendment.

Biochar effect on associations	Compartment	Module	Traits
Newly induced	Rhizosphere	M2	As, Ca, Co, Cr, Cu, K, Mn, Na, NH3-NH4, Ni, NN, pH, Pb, TN, TOC, TP, Zn, MaxD, MD, NetA, P, SA, SRL, V, ß-Glu, Phos
		M3	TP, NN, V
	Rhizoplane	M2	NN
		M3	pH, MaxD, ß-Glu
Maintained	Rhizosphere	M3	As, Ca, Co, Cr, Cu, K, Mn, Na, NH3-NH4, Ni, pH, Pb, TN, TOC, Zn, MaxD, MD, NetA, P, SA, SRL, V, Phos
Missed	Rhizosphere	M4	ß-Glu, Phos

Specifically, in the rhizoplane, among the 4 new biochar-induced associations, one was found between the microbial module M2 and NN, while the others regard the microbial module M3 and MaxD/pH/ ß-Glu (Table 3 and Table S8).

In the rhizosphere, 23 associations were maintained (soil parameters: As, Ca, Co, Cr, Cu, K, Mn, Na, NH_3_-NH_4_, Ni, pH, Pb, TN, TOC, Zn; root features: MaxD, MD, NetA, P, SA, SRL, V; enzymes: Phos) and 3 new induced after biochar amendment (soil parameters: TP, NN; root features: V) among the microbial module M3, soil/root features and enzymes (Table 3 and Table S8). In the same compartment, other 26 new associations were induced among the microbial module M2 and soil/root features (soil parameters: As, Ca, Co, Cr, Cu, K, Mn, Na, NH_3_-NH_4_, Ni, NN, pH, Pb, TN, TOC, TP, Zn; root features: MaxD, MD, NetA, P, SA, SRL, V; enzymes: ß-Glu, Phos) and 2 association, among M4 and ß-Glu/Phos, were contrary missed by biochar treatment (Table 3 and Table S8).

## Discussion

This study provides deep knowledge about the role of biochar amendment in modulating root’s interactions in non-urban and peri-urban soils of two forestry sites of Campobasso (Italy), evidencing shifts in root morphology, soil zymography, microbial community composition, and network.

According to our previous study^11^, specific root adaptive growing strategies were adopted by *Q. cerris* L. in the two soils: in natural soil, root tended to increase the total length and the number of tips, investing in an efficient nutrient foraging, whereas in peri-urban soil, roots diameter and structural robustness were preferentially increased in response to greater nutrient and carbon availability.

These two contrasting strategies could be related to different soil characteristics: peri-urban soils displayed higher pH, total organic carbon (TOC), total nitrogen (TN), and ammoniacal nitrogen (NH_3_-NH_4_) levels compared to non-urban soils. The peri-urban soils contained also the highest concentrations of certain metal(loid)s (Co, As, Ni, Pb) and elements (K, Na). Also the extracellular enzyme activities (ß-Glu, Phos and LAP) were higher in the peri-urban soils, than in non-urban, particularly on the rhizoplane compartment. In this compartment, the organic compounds on the root surface could be easily degraded^71^, making the root–soil interface a critical hotspot for enzyme activity and nutrient cycling^72–74^.

The clear distinction between the rhizosphere and rhizoplane prokaryotic communities in the two soils could likely reflecting soil chemical differences, with *Firmicutes* and *Verrucomicrobiota* were more abundant in non-urban soils, whereas *Acidobacteriota* and *Actinobacteriota* enriched in peri-urban soils. Furthermore, rhizosphere communities exhibited greater heterogeneity, whereas a limited number of bacterial taxa dominated the rhizoplane communities.

The rhizoplane-enriched genera included taxa involved in nitrogen fixation, phosphate solubilization, and plant growth promotion. For instance, members of the *Allorhizobium-Neorhizobium-Pararhizobium-Rhizobium* group are well-known diazotrophs that enhance nitrogen availability to plants^75–77^. Similarly, *Massilia* spp. are able to solubilize recalcitrant phosphate sources, thereby improving phosphorus uptake^75,78^. *Novosphingobium* has been widely reported as a root-associated genus; previous studies showed higher abundance in the rhizoplane than in the rhizosphere and bulk soil of various plants^77–80^. *Flavobacterium* and *Pseudomonas* are commonly described as rhizoplane-associated bacteria with plant growth-promoting traits, including biofilm formation, pathogen suppression, and production of bioactive metabolite^81–85^.

In contrast, the rhizosphere communities were enriched with genera typically associated with soil ecological processes, such as *Bradyrhizobium*, a well-known nitrogen-fixing bacterium^66,86^ and *Bryobacter*, a PGPB involved in the decomposition of organic matter^87^, together with other phyla associated with healthy soils (ex. *Candidatus Udaeobacter* and *Candidatus Xiphinematobacter*)^88–91^.

Overall, this pattern reflects the soil–root continuum, where microbial diversity decreases from bulk soil to the rhizoplane due to increasing plant-mediated selection pressure. Consequently, specific taxa are progressively enriched near the root surface, while others are excluded^92^. In this framework, bulk soil acts as a microbial reservoir, the rhizosphere as a site of microbial proliferation, and the rhizoplane as a selective boundary towards the root endosphere^92–94^.

Analysing the effect of short-term biochar application, it did not substantially alter the chemical characteristics, increasing only nitric nitrogen content. However, biochar amendment results able to reinforce pre-existent patterns in both non-urban and peri-urban soils, influencing extracellular enzymatic activity, *Q. cerris* root morphology, microbial community composition and network. The negligible effect of biochar on the main soil chemical parameters, such as pH, TOC and electric conductance, has been previously shown in forest soils^95–98^, mainly attributed to biochar feedstock, and pyrolysis process, in addition to those of the local environment^99,100^.

However, in non-urban soils, biochar tended to increase root length and the number of root tips, promoting foraging-type growth. In peri-urban soils, where roots were already thicker and more robust, biochar maintained a conservative growth pattern. Thus, in both soils, biochar acted as a general root growth enhancer, amplifying existing adaptive strategies rather than altering them^101–103^ These root growing patterns may contribute to the formation of localized areas in which the extracellular enzymatic activity and microbial communities were differentially distributed in the two soils and root compartments.

Consistent with data reported in literature, in the rhizosphere, biochar amendment was found to increase ß-glucosidase^104,105^ activity in both soil types and acidic phosphatase activity^106^ only in non-urban soil. Furthermore, *Armatimonadota* abundance decreased, whereas *Patescibacteria* increased^20,107–110^ in non-urban soils amended with biochar, while a significant reduction in the abundance of *Firmicutes* was observed in the rhizosphere of biochar-treated peri-urban soils. However, although biochar can influence nutrient availability and, directly or indirectly, the abundance of different microbial taxa, no universal or unidirectional patterns have been observed^111,112^.

Moving from phylum to genus level, specific taxa were associated with biochar amendment: *Inquilinus* in non-urban soils, and of *BIrii41* and *MND1* in peri-urban soils. *Inquilinus* is known for phosphate solubilization and biocontrol capability^113,114^, while, *MND1* is a genus within the *Nitrosomonadaceae* family which includes nitrifying, N-fixing, and cellulose-decomposing bacteria^115^. The taxon *BIrii41* was identified as an indicator of organic fertilizer treatments in a research evaluating different agricultural management systems, exhibiting plant growth-promoting attributes^102,116^.

Hence, it is likely that biochar treatment could provide a more suitable environment for “plant-friendly” bacteria in the soil, which in turn promote plant growth and development^115^ and a clear structural reorganization of the microbial network in the two sites.

Biochar significantly increased degree centrality in non-urban soils and reduced the network connectivity in peri-urban soils. This pattern suggested that, under favourable conditions such as those found in non-urban soils, biochar may promote cooperative interactions among microbial taxa. This effect could be related to the provision of additional microsites and improved nutrient and moisture retention, which facilitate niche complementarity^117,118^. Conversely, microbial communities in disturbed environments, such as peri-urban soils, are typically less linked and more isolated across ecological niches. Consequently, biochar may favour a subset of opportunistic taxa, thereby reducing the overall network complexity^119^. This effect was most pronounced in the rhizosphere, where biochar more than doubled the number of significant correlations between microbial communities, soil parameters, root features and enzymatic activities. Specifically, biochar amendment was significantly correlated with the genera clustered in microbial module M2 and total phosphorus/root volume/ß -glucosidase and phosphatase enzymatic activity, as well as with module M3 and nitric nitrogen content/ phosphatase enzymatic activity.

The module M2 included some genera related to plant growth-promoting bacteria (PGPB), such as *Azospira*, *Azospirillum* and *Paenibacillus*, which may contribute to the observed increase in root volume^120–122^. Genomic studies have also shown that many species of *Paenibacillus* can solubilize phosphorus, explaining the strong correlation observed between this module and phosphorus availability^121^.

The genera within the module M3, such as *Gemmatiomonas, Pseudolabrys* (hub genus) and *Nitrosospira*, or *Clostridium,* are denitrifiers^123–126^ or nitrate assimilator^127^, therefore capable of using nitrate either as a source of assimilable nitrogen or as a terminal electron acceptor in dissimilatory reduction processes^128^. In this context, biochar—by increasing NO₃⁻ availability in both non-urban and peri-urban soils—may enhance microbial communities involved in nitrate transformation^129,130^.

It is important to highlight that biochar treatment disrupted in the rhizosphere the association between bacteria of the module M4—such as *Acidothermus* and* BIrii41*—and ß -glucosidase/phosphatase enzymatic activities. Both taxa are organic matter decomposer, known to thrive in acidic soils and sensitive to soil moisture content^131^, that could reflect a partial change also in nutrient availability in the rhizosphere.

Biochar also modulated rhizoplane networks inducing new significant correlations: the genera *Burkholderia, Caballeronia and Paraburkholderia* (BCP), clustered in the module M2, were found negatively correlated with nitric nitrogen, while the genera belonging to the module M3, such as *Acidipila*, *Niastella*, and *Phycicoccus*, were positively correlated with pH and maximum root diameter and negatively with phosphatase enzymatic activity.

The first pattern likely reflects a functional reorganization of the microbiome in response to nitrogen enrichment^132–134^; BCP facultative diazotrophs and PGPB — capable of regulating nitrogen availability^135,136^ — may act as a connector between nitrifying, denitrifying and decomposing populations, contributing to the stabilization of the nitrogen cycle^137,138^. In this scenario, biochar may contribute to the development of a more structured and interconnected microbial network, in which BCP genera may act as important hubs potentially contributing to nitrogen cycling under nutrient-rich conditions.

About the second pattern, despite biochar treatment did not appear to affect soil pH — likely due to the short experimental timeframe — it may still generate microsites with heterogeneous nutrient availability^117,118^. These microscale variations could shift microbial community interactions and lead to new correlations between microbial network structure and soil/root parameters/enzymes, which could help sustain essential ecosystem functions.

## Conclusions

The present study sheds light on the short-term effect of biochar on the composition and spatial distribution of the bacterial communities in the rhizosphere and on the rhizoplane of *Q. cerris* seedlings growing in non-urban and peri-urban soils. In detail, in both soils, biochar enhances root-microbe interactions, probably generating microsites with heterogeneous nutrient availability. However, in the rhizosphere, it induces a redistribution of keystone (hub) taxa, mostly based on less abundant species (relative abundance < 0.5%), while, the microbiota remained more stable on the rhizoplane. Biochar promotes also root growth, inducing adaptive foraging strategies in non-urban soil and conservative strategies in peri-urban soil. However, some limitations of this study should be considered. In particular, the narrow time frame of the experiment, combined with the use of a single plant species in controlled rhizobox systems, may have restricted the overall interpretation of the findings, especially for manifold settings such as peri-urban ones. Further research is needed to fully understand biochar’s progressive (medium- and long-term) impact on the soil physicochemical properties and on the stability of interactions that occur over time, by using also other plant species. Functional approaches, such as meta transcriptomics combined with plant labelling, could allow for detailed investigations of how and where root-microbe interactions are functionally reshaped by biochar.

Overall, and in the context of sustainable soil management, this research would provide a better understanding of how biochar can support the ecological restoration and resilience of degraded peri-urban systems.

## Supplementary Information

Below is the link to the electronic supplementary material.

Supplementary Information 1.

## Data Availability

The sequencing datasets generated during and/or analysed during the current study are available in the NCBI Sequence Read Archive (SRA) and are available under the accession number PRJNA1368845.
