# Supplementary material for: Short-term effects of biochar amendment on root–microbe interactions in natural and peri-urban soils
Source: Sci Rep. 2026 Apr 9;16:16769. doi: 10.1038/s41598-026-46789-z (PMC13222878; doi:10.1038/s41598-026-46789-z)
Supplement: Supplementary file 1 — Supplementary Information 1. [file 41598_2026_46789_MOESM1_ESM.docx]

**Supplementary Materials**

**
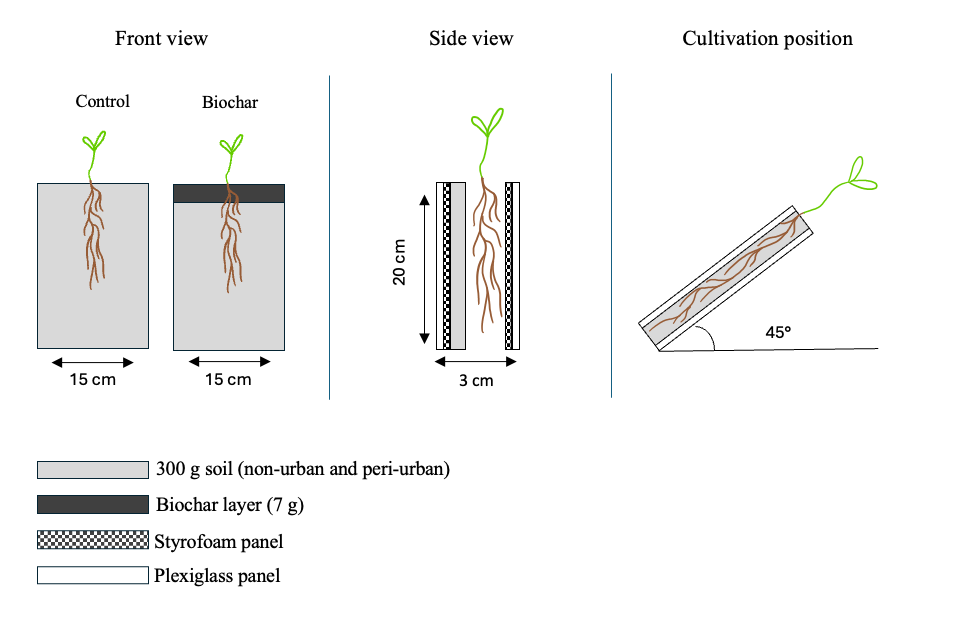
**

Figure S1. Schematic representation of the rhizobox setup and plant growing position.


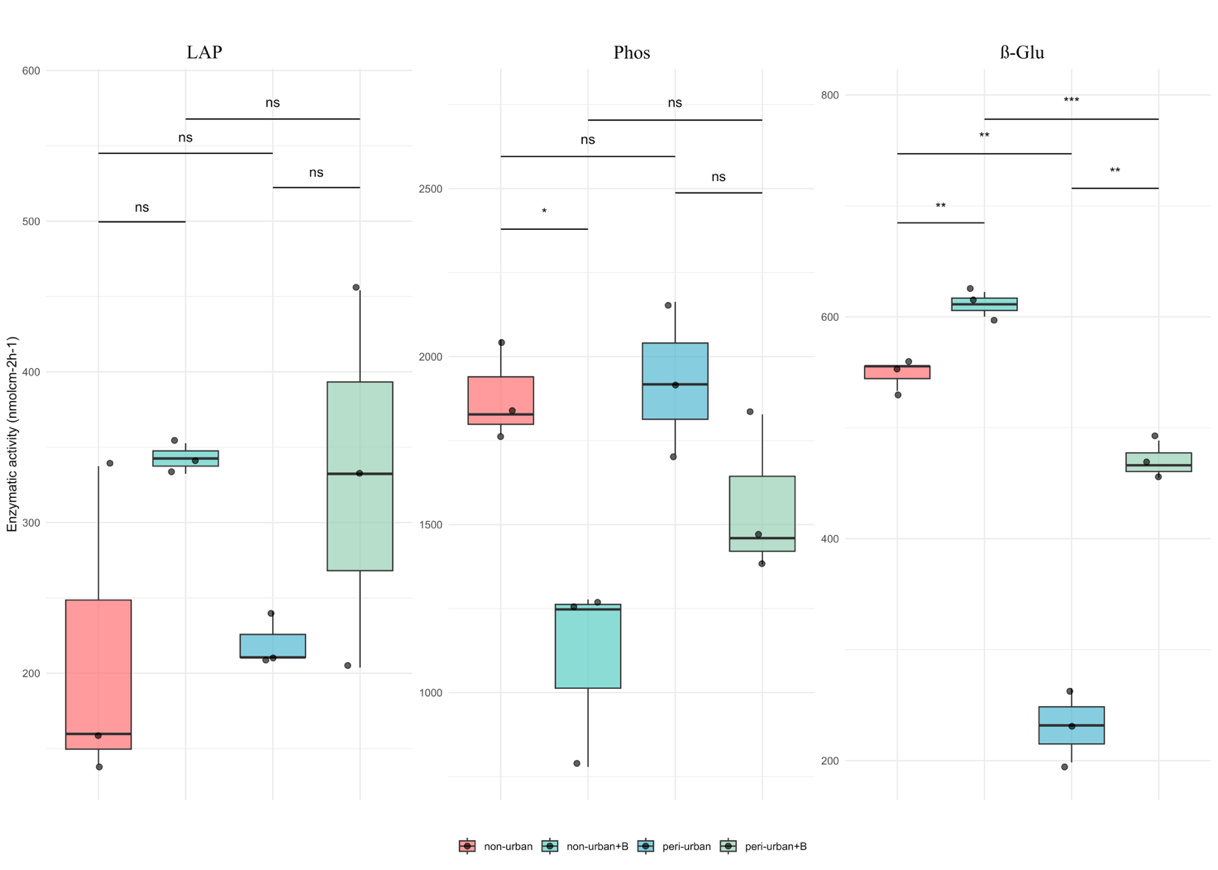


Figure S2. Boxplot of the enzymatic activity revealed on the entire rhizobox area of the four experimental groups [non-urban, non-urban + biochar (B), peri-urban, peri-urban +B]. Tested enzymes: ß-Glu: ß-glucosidase, Phos: phosphatases and LAP: leucine amino-peptidase expressed as nmolcm^-2^h^-1^.





Figure S3. Rhizoplane and rhizosphere enzymatic activity in the four experimental groups [non-urban, non-urban + biochar (B), peri-urban, peri-urban +B]. A) The image displays how rhizoplane and rhizosphere were selected in the zymograms (16 colors mode- ImageJ). B) The box plot shows the activity (nmolcm^-2^h^-1^) in rhizosphere and on rhizoplane of three selected enzymes: ß-Glu: ß-glucosidase, Phos: phosphatases and LAP: leucine amino-peptidase. C) The hotspot percentage areas of ß-Glu (left side) and Phos (right side) are reported in the on the corresponding zymograms.





Figure S4. Degree and betweenness centrality fold change (%) of rhizosphere and rhizoplane networks under biochar treatment [non-urban *vs* non-urban + biochar (B); peri-urban *vs* peri-urban +B].

Table S1. Chemical properties of biochar and the two selected soils before the experimentTOC - Total Organic Carbon, TN - Total nitrogen, N_NH3-NH4 -_ ammoniacal nitrogen_,_ NN - nitric nitrogen, TP - Total phosphorus, P_av_ - available phosphorus, B - Boron, Na -Sodium, K - Potassium, As - Arsenic, Ca - Calcium, Cd - Cadmium, Cr - Chromium, Co - Cobalt, Cu - Copper, Pb - Lead, Hg - Mercury, Ni - Nickel, Zn – Zinc and Mn - Manganese.

| ***Chemical properties*** | **Biochar** | **Non-urban (T0)** | **Peri-urban (T0)** |
| --- | --- | --- | --- |
| **pH** | 6.11 ± 0.07 | 7.67 ± 0.08 | 8.69 ±0.1 |
| **TOC (%dm)** | 1.14 ± 0.19 | 2.01 ± 0.2 | 1 ±0.01 |
| **TN (%dm)** | 0.09 ± 0.01 | 0.1 ± 0.5 | 0.27 ±0.01 |
| **N_NH3-NH4_ (mg/Kg dm)** | 39.6 ± 16.7 | 13.3± 8.8 | <0.5 |
| **NN (%dm)** | 0.52 ± 0.15 | 1.32 ± 1.47 | 65.6 ±0.1 |
| **TP (% dm)** | 0.01± 0.01 | 0.01 ± 0.01 | 0.14 ±0 |
| **P_av_ (mg/Kg dm)** | <1 | <1 | 636 ±0.1 |
| **B (mg/Kg dm)** | <5 | <5 | 28.6 ±0.1 |
| **Na (mg/Kg dm)** | 132.2 ± 21.5 | 279 ± 110.6 | 663 ±32.4 |
| **K (mg/Kg dm)** | 450± 100 | 3000 ± 49.7 | 8877 ±200 |
| **As (mg/Kg dm)** | 1.9 ± 0.9 | 4.4 ± 0.5 | <1.5 |
| **Ca (mg/Kg dm)** | 1195 ± 110 | 7450 ± 156 | 32186.66 ±120 |
| **Cd (mg/Kg dm)** | <0.35 | <0.35 | <0.35 |
| **Cr (mg/Kg dm)** | 8.9 ± 0.3 | 24 ± 10 | <9 |
| **Co (mg/Kg dm)** | 2.9 ± 0.3 | 10 ± 2.7 | 1.67 ±0.7 |
| **Cu (mg/Kg dm)** | 3.8 ± 0.4 | 20 ± 1.5 | 10.5 ±0.3 |
| **Pb (mg/Kg dm)** | 7.7 ± 0.5 | 15.8 ± 0.7 | 6.2 ±0.2 |
| **Hg (mg/Kg dm)** | <0.3 | <0.3 | <0.3 |
| **Ni (mg/Kg dm)** | 4.5 ± 1.3 | 22.7 ± 6.7 | 7.63 ±0.3 |
| **Zn (mg/Kg dm)** | 18.6 ± 11.3 | 49 ± 15.3 | <1 |
| **Mn (mg/Kg dm)** | 430 ± 34.6 | 850 ± 115.4 | 212 ±11.5 |

Table S2. Mean relative abundances of the prokaryotic phyla retrieved in the rhizosphere and rhizoplane compartments across the four experimental groups [non-urban, non-urban + biochar (B), peri-urban, peri-urban +B].

|  | **Rhizoplane** | | | |  | **Rhizosphere** | | | |
| --- | --- | --- | --- | --- | --- | --- | --- | --- | --- |
| **Phyla** | **non-urban** | **peri-urban** | **non-urban + B** | **peri-urban + B** |  | **non-urban** | **peri-urban** | **non-urban + B** | **peri-urban + B** |
| *Firmicutes* | 3.38 | 6.50 | 2.76 | 7.98 |  | 37.12 | 36.61 | 36.99 | 30.51 |
| *Proteobacteria* | 74.80 | 59.18 | 72.47 | 65.41 |  | 28.13 | 30.10 | 23.71 | 33.18 |
| *Bacteroidota* | 12.17 | 16.88 | 15.89 | 12.39 |  | 6.54 | 4.60 | 8.25 | 5.60 |
| *Verrucomicrobiota* | 0.87 | 2.07 | 0.51 | 0.95 |  | 13.23 | 10.31 | 14.31 | 11.54 |
| *Actinobacteriota* | 5.81 | 8.11 | 6.09 | 10.18 |  | 4.48 | 6.42 | 4.38 | 5.98 |
| *Acidobacteriota* | 0.37 | 0.55 | 0.61 | 0.19 |  | 7.21 | 8.69 | 8.51 | 8.99 |
| *Chloroflexi* | 0.19 | 2.88 | 0.06 | 0.19 |  | 0.85 | 0.61 | 0.70 | 0.77 |
| *Cyanobacteria* | 0.92 | 0.39 | 0.59 | 0.34 |  | 0.23 | 0 | 0.45 | 0.01 |
| *Patescibacteria* | 0.81 | 1.56 | 0.73 | 0.98 |  | 0.40 | 0.00 | 0.96 | 0.05 |
| *Myxococcota* | 0.07 | 0.45 | 0.07 | 0.15 |  | 0.36 | 1.09 | 0.34 | 1.51 |
| *Bdellovibrionota* | 0.34 | 1.28 | 0.07 | 1.15 |  | 0.17 | 0.34 | 0.35 | 0.34 |
| *Gemmatimonadota* | 0.06 | 0.04 | 0.02 | 0.02 |  | 0.78 | 0.23 | 0.81 | 0.40 |
| *Desulfobacterota* | 0 | 0 | 0 | 0 |  | 0 | 0.11 | 0 | 0.08 |
| *Fibrobacterota* | 0 | 0 | 0 | 0 |  | 0.16 | 0 | 0 | 0 |
| *Nitrospirota* | 0 | 0 | 0 | 0 |  | 0.05 | 0 | 0.04 | 0 |
| *Deinococcota* | 0.03 | 0 | 0.06 | 0 |  | 0 | 0 | 0 | 0 |
| *RCP2-54* | 0 | 0 | 0 | 0 |  | 0 | 0.02 | 0 | 0.05 |
| *Armatimonadota* | 0.08 | 0 | 0.02 | 0 |  | 0.03 | 0 | 0 | 0 |
| *Planctomycetota* | 0.01 | 0 | 0 | 0 |  | 0.03 | 0 | 0.07 | 0 |

Table S3. Differential abundant bacterial phyla in both rhizosphere and rhizoplane compartments of soil substrates, considering the comparisons: non-urban vs peri-urban, non-urban vs non-urban + Biochar (B), peri-urban vs peri-urban + B. Statistical significance (*p*$\leq$*0.01*) was determined using ANOVA.

| **Compartment** | **Comparison** | **Phylum** | ***p-value*** | **Log2FC** |
| --- | --- | --- | --- | --- |
| **Rhizoplane** | non-urban vs peri-urban | *-* |  | - |
|  | non-urban vs non-urban + B | *-* |  | - |
|  | peri-urban vs peri-urban + B | *-* |  | - |
| **Rhizosphere** | non-urban vs peri-urban | *Gemmatimonadota* | *0.000008* | 1.588 |
|  |  | *Myxococcota* | *0.000106* | -1.67 |
|  |  | *Actinobacteriota* | *0.007523* | -0.507 |
|  | non-urban vs non-urban + B | *Armatimonadota* | *0.003438* | 2.029 |
|  |  | *Patescibacteria* | *0.007269* | -1.078 |
|  | peri-urban vs peri-urban + B | *Firmicutes* | *0.002736* | 0.312 |

Table S4. Mean relative abundances of the prokaryotic genera retrieved in the rhizosphere and rhizoplane compartments across the four experimental groups [non-urban, non-urban + biochar (B), peri-urban, peri-urban +B].

|  | **Rhizoplane** | | | |  | **Rhizosphere** | | | |
| --- | --- | --- | --- | --- | --- | --- | --- | --- | --- |
| **Genus** | **non-urban** | **peri-urban** | **non-urban + B** | **peri-urban + B** |  | **non-urban** | **peri-urban** | **non-urban + B** | **peri-urban + B** |
| *g__0319-6G20* | 0.009 | 0.047 | 0 | 0.009 |  | 0 | 0 | 0.061 | 0 |
| *g__11-24* | 0 | 0.017 | 0 | 0.007 |  | 0 | 0.326 | 0 | 0.352 |
| *g__1959-1* | 0 | 0 | 0 | 0 |  | 0.009 | 0 | 0.021 | 0 |
| *g__37-13* | 0 | 0.058 | 0 | 0.007 |  | 0 | 0 | 0 | 0 |
| *g__67-14* | 0.006 | 0.037 | 0 | 0.012 |  | 0.057 | 0.298 | 0.036 | 0.248 |
| *g__A21b* | 0 | 0 | 0 | 0 |  | 0.016 | 0 | 0.039 | 0 |
| *g__Achromobacter* | 0.186 | 0.071 | 0.012 | 0.141 |  | 0.010 | 0.021 | 0.011 | 0.043 |
| *g__Acidibacter* | 0 | 0 | 0 | 0 |  | 0 | 0.187 | 0 | 0.223 |
| *g__Acidipila* | 0.008 | 0 | 0.021 | 0 |  | 0.451 | 0 | 0.467 | 0 |
| *g__Acidothermus* | 0 | 0 | 0 | 0 |  | 0.036 | 0 | 0.100 | 0 |
| *g__Acidovorax* | 0.877 | 2.170 | 0.358 | 0.728 |  | 0.288 | 0.221 | 0.223 | 0.319 |
| *g__Acinetobacter* | 0.578 | 1.274 | 0.048 | 1.377 |  | 0.009 | 0.298 | 0.002 | 0.127 |
| *g__Actinoallomurus* | 0.018 | 0 | 0.010 | 0 |  | 0.111 | 0 | 0.070 | 0 |
| *g__Actinoplanes* | 0.395 | 0.390 | 0 | 0.141 |  | 0.011 | 0 | 0 | 0 |
| *g__Actinopolymorpha* | 0 | 0 | 0 | 0 |  | 0.010 | 0.008 | 0 | 0.036 |
| *g__Adhaeribacter* | 0 | 0 | 0 | 0.018 |  | 0.011 | 0.246 | 0.022 | 0.166 |
| *g__ADurb.Bin063-1* | 0 | 0 | 0.010 | 0 |  | 0.353 | 0 | 0.663 | 0 |
| *g__Aeromonas* | 0 | 0.100 | 0.007 | 0.193 |  | 0 | 0.037 | 0 | 0.003 |
| *g__Aetherobacter* | 0.016 | 0 | 0 | 0 |  | 0.073 | 0 | 0.024 | 0 |
| *g__Agromyces* | 0 | 0.233 | 0 | 0.172 |  | 0 | 0.446 | 0 | 0.359 |
| *g__AKIW781* | 0 | 0 | 0.006 | 0 |  | 0.017 | 0 | 0.012 | 0 |
| *g__Algoriphagus* | 0 | 0.118 | 0.005 | 0 |  | 0 | 0 | 0 | 0 |
| *g__Alkanibacter* | 0 | 0 | 0.106 | 0 |  | 0.015 | 0 | 0.032 | 0 |
| *g__Allorhizobium-Neorhizobium-Pararhizobium-Rhizobium* | 7.807 | 8.052 | 6.701 | 9.693 |  | 0.649 | 0.543 | 0.839 | 0.642 |
| *g__alphaI_cluster* | 0.021 | 0 | 0 | 0 |  | 0.027 | 0 | 0.031 | 0 |
| *g__Alsobacter* | 0.159 | 0 | 0.233 | 0 |  | 0 | 0 | 0.011 | 0 |
| *g__Altererythrobacter* | 0 | 0.113 | 0 | 0.025 |  | 0 | 0.018 | 0 | 0.024 |
| *g__Ammoniphilus* | 0 | 0 | 0 | 0 |  | 0.003 | 0 | 0.036 | 0 |
| *g__Anaeromyxobacter* | 0 | 0 | 0.006 | 0 |  | 0.006 | 0 | 0.022 | 0 |
| *g__Aquabacterium* | 0.025 | 0.049 | 0 | 0.072 |  | 0 | 0.007 | 0 | 0 |
| *g__Aquicella* | 0 | 0 | 0 | 0.029 |  | 0 | 0 | 0 | 0.033 |
| *g__Aquisphaera* | 0 | 0 | 0 | 0 |  | 0.004 | 0 | 0.032 | 0 |
| *g__Archangium* | 0 | 0 | 0 | 0 |  | 0 | 0.163 | 0 | 0.212 |
| *g__Arcticibacter* | 0.055 | 0.361 | 0.067 | 0.059 |  | 0 | 0.151 | 0 | 0.138 |
| *g__Arenimonas* | 0 | 0.089 | 0 | 0.016 |  | 0 | 0.018 | 0 | 0.024 |
| *g__Asanoa* | 0 | 0 | 0 | 0 |  | 0 | 0 | 0 | 0.056 |
| *g__Asticcacaulis* | 0.493 | 0.474 | 0.511 | 0.365 |  | 0.297 | 0 | 0.389 | 0.007 |
| *g__Aurantisolimonas* | 0 | 0 | 0 | 0 |  | 0.023 | 0 | 0.008 | 0 |
| *g__Azoarcus* | 0 | 0.146 | 0 | 0.014 |  | 0 | 0.151 | 0 | 0.238 |
| *g__Azohydromonas* | 0.262 | 0.120 | 0 | 0.126 |  | 0.029 | 0.004 | 0 | 0.006 |
| *g__Azospira* | 0 | 0 | 0 | 0.008 |  | 0.042 | 0.017 | 0.014 | 0.068 |
| *g__Azospirillum* | 0.210 | 0.080 | 0.251 | 0.066 |  | 0.290 | 0.165 | 0.127 | 0.141 |
| *g__Bacillus* | 2.144 | 4.738 | 2.036 | 6.982 |  | 20.490 | 27.346 | 12.112 | 21.138 |
| *g__bacteriap25* | 0 | 0.007 | 0 | 0 |  | 0 | 0.428 | 0 | 0.633 |
| *g__Bacteriovorax* | 0.102 | 0.201 | 0.072 | 0.071 |  | 0.096 | 0 | 0.258 | 0.010 |
| *g__Bacteroides* | 0.020 | 0 | 0 | 0.009 |  | 0.009 | 0.016 | 0 | 0 |
| *g__Bauldia* | 0 | 0.033 | 0 | 0 |  | 0 | 0.059 | 0 | 0.047 |
| *g__Bdellovibrio* | 0.179 | 0.953 | 0 | 0.928 |  | 0.058 | 0.302 | 0.023 | 0.242 |
| *g__BIrii41* | 0 | 0 | 0 | 0 |  | 0.051 | 0.034 | 0.016 | 0.096 |
| *g__BIyi10* | 0 | 0 | 0 | 0.010 |  | 0 | 0.018 | 0 | 0.037 |
| *g__Bosea* | 0.187 | 0.352 | 0.106 | 0.072 |  | 0.007 | 0 | 0.010 | 0 |
| *g__Bradyrhizobium* | 0.249 | 0.444 | 0.313 | 0.262 |  | 1.931 | 1.271 | 1.782 | 1.050 |
| *g__Brevibacillus* | 0.005 | 0 | 0 | 0 |  | 0.061 | 0 | 0.011 | 0 |
| *g__Brevundimonas* | 0 | 0 | 0.058 | 0 |  | 0 | 0 | 0 | 0 |
| *g__Bryobacter* | 0.074 | 0.074 | 0.092 | 0.018 |  | 1.500 | 0.925 | 1.775 | 1.028 |
| *g__Burkholderia-Caballeronia-Paraburkholderia* | 4.803 | 0.461 | 11.279 | 0.398 |  | 1.671 | 0.067 | 1.973 | 0.028 |
| *g__C0119* | 0.035 | 0 | 0.035 | 0 |  | 0.180 | 0 | 0.240 | 0 |
| *g__Caenimonas* | 0.071 | 0 | 0.065 | 0 |  | 0.214 | 0 | 0.123 | 0.010 |
| *g__Candidatus_Endoecteinascidia* | 0 | 0 | 0 | 0 |  | 0.014 | 0 | 0.017 | 0 |
| *g__Candidatus_Koribacter* | 0.039 | 0 | 0.092 | 0 |  | 1.059 | 0 | 1.430 | 0 |
| *g__Candidatus_Paracaedibacter* | 0.003 | 0 | 0.004 | 0 |  | 0.009 | 0 | 0.023 | 0 |
| *g__Candidatus_Solibacter* | 0.039 | 0.032 | 0.017 | 0 |  | 0.798 | 0.238 | 1.265 | 0.350 |
| *g__Candidatus_Udaeobacter* | 0.290 | 0.296 | 0.133 | 0.059 |  | 9.376 | 4.896 | 9.490 | 4.701 |
| *g__Candidatus_Xiphinematobacter* | 0.090 | 0.239 | 0.057 | 0.120 |  | 1.343 | 4.226 | 1.423 | 5.260 |
| *g__Catellatospora* | 0 | 0.016 | 0 | 0.014 |  | 0 | 0.081 | 0 | 0.130 |
| *g__Caulobacter* | 0.277 | 0.314 | 0.036 | 0.387 |  | 0.012 | 0.025 | 0 | 0.023 |
| *g__CCD24* | 0 | 0 | 0 | 0 |  | 0 | 0.141 | 0 | 0.158 |
| *g__Cellulomonas* | 0.078 | 0.158 | 0.043 | 0.023 |  | 0.014 | 0 | 0.009 | 0.006 |
| *g__Cellvibrio* | 0.064 | 2.972 | 0.337 | 2.085 |  | 0.006 | 0.291 | 0.013 | 0.441 |
| *g__Chitinimonas* | 0.005 | 0.266 | 0 | 0.146 |  | 0 | 0.011 | 0 | 0.028 |
| *g__Chitinophaga* | 1.941 | 0.332 | 2.475 | 0.754 |  | 0.367 | 0.051 | 0.549 | 0.108 |
| *g__Chryseobacterium* | 1.161 | 0 | 0 | 0 |  | 0 | 0 | 0 | 0 |
| *g__Chryseolinea* | 0 | 0.075 | 0 | 0.051 |  | 0 | 0.358 | 0 | 0.375 |
| *g__Chthoniobacter* | 0.095 | 0.095 | 0.011 | 0.076 |  | 0.277 | 0.669 | 0.156 | 0.664 |
| *g__Citrifermentans* | 0 | 0 | 0 | 0 |  | 0 | 0.115 | 0 | 0.085 |
| *g__CL500-29_marine_group* | 0 | 0 | 0 | 0 |  | 0.020 | 0.009 | 0.015 | 0.017 |
| *g__Clostridium_sensu_stricto_1* | 0 | 0 | 0 | 0 |  | 0.045 | 0 | 0.011 | 0 |
| *g__Clostridium_sensu_stricto_10* | 0.012 | 0 | 0 | 0.021 |  | 1.154 | 1.486 | 0 | 2.434 |
| *g__Clostridium_sensu_stricto_12* | 0 | 0 | 0 | 0 |  | 0.030 | 0 | 0.049 | 0 |
| *g__Clostridium_sensu_stricto_13* | 0 | 0.007 | 0 | 0.014 |  | 0.242 | 0.213 | 0.092 | 0.950 |
| *g__Clostridium_sensu_stricto_5* | 0.010 | 0 | 0 | 0 |  | 0 | 0 | 0.034 | 0 |
| *g__Clostridium_sensu_stricto_8* | 0 | 0 | 0 | 0 |  | 0.043 | 0.088 | 0 | 0.150 |
| *g__Clostridium_sensu_stricto_9* | 0.042 | 0 | 0 | 0 |  | 0.697 | 0 | 0.041 | 0 |
| *g__Cohnella* | 0.011 | 0.013 | 0 | 0 |  | 0.061 | 0.190 | 0.132 | 0.146 |
| *g__Comamonas* | 0.145 | 1.307 | 0.519 | 0.521 |  | 0.053 | 0.181 | 0.095 | 0.201 |
| *g__Cupriavidus* | 0.228 | 0.209 | 0.136 | 0.091 |  | 0.043 | 0.004 | 0.038 | 0.007 |
| *g__Curtobacterium* | 0.017 | 0 | 0.154 | 0.195 |  | 0 | 0 | 0 | 0 |
| *g__Curvibacter* | 0.040 | 0.162 | 0 | 0.061 |  | 0.261 | 0.181 | 0.037 | 0.320 |
| *g__Cytophaga* | 0.083 | 0.017 | 0.096 | 0.009 |  | 0.107 | 0 | 0.057 | 0 |
| *g__D05-2* | 0 | 0.011 | 0 | 0.006 |  | 0 | 0.061 | 0 | 0.048 |
| *g__Dactylosporangium* | 0 | 0.012 | 0 | 0 |  | 0.229 | 0.160 | 0.261 | 0.172 |
| *g__Dechlorobacter* | 0 | 0 | 0 | 0 |  | 0 | 0 | 0 | 0.035 |
| *g__Dechloromonas* | 0.094 | 0.203 | 0 | 0.391 |  | 0.114 | 0.653 | 0.077 | 0.565 |
| *g__Dechlorosoma* | 0 | 0 | 0 | 0.088 |  | 0 | 0.170 | 0 | 0.204 |
| *g__Defluviimonas* | 0.018 | 0.019 | 0 | 0.195 |  | 0 | 0 | 0 | 0 |
| *g__Deinococcus* | 0.037 | 0 | 0.066 | 0 |  | 0 | 0 | 0 | 0 |
| *g__Devosia* | 0.264 | 2.164 | 0.131 | 0.645 |  | 0.015 | 0.391 | 0 | 0.335 |
| *g__Diaphorobacter* | 0 | 0 | 0 | 0.064 |  | 0 | 0 | 0 | 0.009 |
| *g__Domibacillus* | 0 | 0.034 | 0 | 0.146 |  | 0 | 0.043 | 0 | 0.064 |
| *g__Dongia* | 0 | 0.082 | 0 | 0.028 |  | 0 | 0.535 | 0 | 0.463 |
| *g__Duganella* | 0.006 | 0.070 | 0.059 | 0 |  | 0 | 0 | 0 | 0.004 |
| *g__Dyadobacter* | 0.390 | 1.669 | 0.044 | 0.631 |  | 0.001 | 0.006 | 0.002 | 0.021 |
| *g__Dyella* | 0.127 | 0 | 0.035 | 0 |  | 0.237 | 0.008 | 0.052 | 0 |
| *g__Edaphobacter* | 0.011 | 0.019 | 0.062 | 0 |  | 0.266 | 0.007 | 0.341 | 0.045 |
| *g__Edaphobaculum* | 0.014 | 0 | 0.007 | 0 |  | 0.028 | 0 | 0.070 | 0 |
| *g__Ellin516* | 0 | 0 | 0.057 | 0 |  | 0.069 | 0 | 0.234 | 0 |
| *g__Ellin517* | 0.038 | 0.062 | 0.042 | 0.021 |  | 0.467 | 0.161 | 0.665 | 0.203 |
| *g__Enterobacter* | 13.400 | 0.024 | 1.460 | 1.793 |  | 1.265 | 0.022 | 1.450 | 0.008 |
| *g__Enterobacteriaceae* | 0.065 | 0 | 0 | 0 |  | 0 | 0 | 0 | 0 |
| *g__env.OPS_17* | 0.008 | 0.023 | 0 | 0 |  | 0.256 | 0.023 | 0.388 | 0.011 |
| *g__Erwinia* | 0.073 | 0 | 0 | 0 |  | 0 | 0 | 0 | 0 |
| *g__Ferribacterium* | 0 | 0 | 0 | 0.008 |  | 0 | 0.134 | 0 | 0.536 |
| *g__Ferrovibrio* | 0 | 0.026 | 0 | 0.014 |  | 0 | 0.042 | 0 | 0.030 |
| *g__Ferruginibacter* | 0 | 0 | 0 | 0 |  | 0.055 | 0 | 0.009 | 0 |
| *g__Filimonas* | 0.015 | 0 | 0.084 | 0.005 |  | 0 | 0 | 0.001 | 0 |
| *g__Fimbriimonadaceae* | 0.087 | 0 | 0.023 | 0 |  | 0.038 | 0 | 0.009 | 0 |
| *g__Flavihumibacter* | 0 | 0.201 | 0 | 0 |  | 0 | 0.025 | 0 | 0 |
| *g__Flavisolibacter* | 0.195 | 0.060 | 0.047 | 0.039 |  | 0.643 | 0.269 | 0.439 | 0.285 |
| *g__Flavitalea* | 0 | 0.036 | 0 | 0.012 |  | 0 | 0.246 | 0 | 0.226 |
| *g__Flavobacterium* | 6.483 | 10.236 | 8.634 | 8.749 |  | 0.151 | 0.267 | 0.037 | 0.277 |
| *g__Fluviicola* | 0.148 | 0.188 | 0.124 | 0.702 |  | 0 | 0.049 | 0 | 0.026 |
| *g__Fonticella* | 0.006 | 0 | 0 | 0 |  | 0.125 | 0 | 0.080 | 0 |
| *g__Gaiella* | 0.037 | 0.038 | 0.007 | 0 |  | 0.186 | 0.084 | 0.094 | 0.069 |
| *g__Gemmatimonas* | 0.064 | 0.014 | 0.022 | 0 |  | 0.638 | 0.063 | 0.711 | 0.204 |
| *g__Gemmobacter* | 0 | 0.791 | 0.029 | 0.602 |  | 0 | 0.009 | 0 | 0.007 |
| *g__Glutamicibacter* | 0 | 0 | 0 | 0.250 |  | 0 | 0 | 0 | 0 |
| *g__Granulicella* | 0.008 | 0 | 0.144 | 0 |  | 0.180 | 0 | 0.283 | 0 |
| *g__Haliangium* | 0 | 0.047 | 0 | 0.023 |  | 0.105 | 0.032 | 0.173 | 0.175 |
| *g__Herbaspirillum* | 2.581 | 1.283 | 7.318 | 0.331 |  | 0.111 | 0 | 0.122 | 0.006 |
| *g__Herpetosiphon* | 0 | 2.802 | 0.010 | 0.168 |  | 0 | 0.165 | 0 | 0.157 |
| *g__Hirschia* | 0 | 0.031 | 0 | 0.023 |  | 0 | 0.039 | 0 | 0.018 |
| *g__Hydrogenophaga* | 0 | 2.561 | 0.063 | 0.851 |  | 0 | 0.673 | 0 | 0.763 |
| *g__Hyphomicrobium* | 0 | 0 | 0 | 0 |  | 0 | 0.044 | 0 | 0.033 |
| *g__Ideonella* | 0.418 | 0 | 0.202 | 0 |  | 0.422 | 0 | 0.370 | 0 |
| *g__Ilumatobacter* | 0.005 | 0 | 0 | 0 |  | 0 | 0.016 | 0 | 0.022 |
| *g__IMCC26256* | 0 | 0.010 | 0 | 0 |  | 0.030 | 0.097 | 0.024 | 0.096 |
| *g__Inhella* | 0 | 0.181 | 0.014 | 0 |  | 0 | 0.015 | 0 | 0 |
| *g__Inquilinus* | 0 | 0.118 | 0 | 0.027 |  | 0.003 | 0.037 | 0.024 | 0.028 |
| *g__IS-44* | 0 | 0 | 0 | 0 |  | 0 | 0.041 | 0 | 0.063 |
| *g__Jatrophihabitans* | 0.031 | 0 | 0.033 | 0 |  | 0 | 0 | 0.048 | 0 |
| *g__JG30-KF-CM45* | 0 | 0.041 | 0 | 0.013 |  | 0 | 0.194 | 0 | 0.274 |
| *g__JGI_0001001-H03* | 0 | 0 | 0 | 0 |  | 0.052 | 0 | 0.076 | 0 |
| *g__Kapabacteriales* | 0.054 | 0.018 | 0.002 | 0 |  | 0.063 | 0 | 0.035 | 0.008 |
| *g__KD4-96* | 0.013 | 0.045 | 0 | 0 |  | 0.148 | 0.076 | 0.046 | 0.090 |
| *g__KF-JG30-B3* | 0 | 0.007 | 0 | 0 |  | 0.075 | 0.301 | 0.034 | 0.409 |
| *g__KI89A_clade* | 0 | 0 | 0 | 0 |  | 0.004 | 0.046 | 0.002 | 0.061 |
| *g__Kosakonia* | 0.025 | 0.122 | 0 | 1.123 |  | 0 | 0.003 | 0 | 0 |
| *g__Kribbella* | 0.020 | 0.227 | 0 | 0.101 |  | 0.222 | 1.795 | 0.174 | 1.554 |
| *g__Ktedonobacter* | 0.014 | 0 | 0 | 0 |  | 0.099 | 0.011 | 0.092 | 0.003 |
| *g__Labrys* | 0 | 0 | 0 | 0 |  | 0.006 | 0 | 0.015 | 0 |
| *g__Lacibacter* | 0 | 1.247 | 0.031 | 0.066 |  | 0 | 0.018 | 0 | 0.085 |
| *g__Lactobacillus* | 0 | 0 | 0.005 | 0.003 |  | 0.010 | 0.001 | 0.018 | 0.016 |
| *g__Lacunisphaera* | 0 | 0.133 | 0 | 0.058 |  | 0 | 0 | 0 | 0.031 |
| *g__Legionella* | 0.017 | 0 | 0 | 0 |  | 0.122 | 0.047 | 0.003 | 0.042 |
| *g__Leifsonia* | 0 | 0 | 0.082 | 0 |  | 0 | 0 | 0 | 0 |
| *g__Limnobacter* | 0 | 0.149 | 0 | 0.048 |  | 0 | 0.369 | 0 | 0.298 |
| *g__Luedemannella* | 0 | 0.051 | 0 | 0 |  | 0.004 | 0.372 | 0.018 | 0.316 |
| *g__Luteibacter* | 0 | 0 | 0 | 0.016 |  | 0.011 | 0 | 0.023 | 0 |
| *g__Luteolibacter* | 0.136 | 0.617 | 0.125 | 0.332 |  | 0 | 0.007 | 0.006 | 0.043 |
| *g__Lutispora* | 0 | 0 | 0 | 0 |  | 0.010 | 0.002 | 0.009 | 0.015 |
| *g__LWQ8* | 0.322 | 0.346 | 0.375 | 0.147 |  | 0.314 | 0 | 0.858 | 0 |
| *g__Lysinibacillus* | 0 | 0.050 | 0 | 0.028 |  | 0 | 0.115 | 0 | 0.051 |
| *g__Lysobacter* | 0.226 | 0.112 | 0.040 | 0.235 |  | 0.190 | 0.103 | 0.133 | 0.079 |
| *g__Magnetospirillum* | 0 | 0 | 0 | 0 |  | 0.023 | 0.032 | 0 | 0.046 |
| *g__Massilia* | 8.167 | 3.335 | 14.036 | 1.646 |  | 2.115 | 1.304 | 2.049 | 0.597 |
| *g__MB-A2-108* | 0 | 0.032 | 0 | 0.003 |  | 0 | 0.083 | 0 | 0.064 |
| *g__Mesorhizobium* | 0.103 | 0 | 0.347 | 0.018 |  | 0.105 | 0.219 | 0.064 | 0.201 |
| *g__Methylobacillus* | 0 | 0.432 | 0.030 | 0.093 |  | 0 | 0.044 | 0 | 0.032 |
| *g__Methylobacterium-Methylorubrum* | 0.084 | 0.016 | 0.685 | 0.013 |  | 0.056 | 0 | 0.030 | 0 |
| *g__Methylophilus* | 2.181 | 0.682 | 2.243 | 0.293 |  | 0.498 | 0.158 | 0.330 | 0.056 |
| *g__Methylorosula* | 0.090 | 0 | 0.126 | 0 |  | 0.129 | 0 | 0.141 | 0 |
| *g__Methylotenera* | 1.097 | 1.013 | 1.405 | 1.509 |  | 0.160 | 3.771 | 0.256 | 3.566 |
| *g__Methyloversatilis* | 0 | 0.256 | 0.063 | 0.256 |  | 0 | 0.052 | 0 | 0.062 |
| *g__Methylovorus* | 0.007 | 0.013 | 0.006 | 0.051 |  | 0 | 0 | 0 | 0 |
| *g__Microlunatus* | 0 | 0 | 0 | 0 |  | 0.072 | 0 | 0.034 | 0 |
| *g__Micromonospora* | 0 | 0.024 | 0 | 0.047 |  | 0 | 0.530 | 0 | 0.579 |
| *g__Microvirga* | 0.042 | 0.240 | 0 | 0.280 |  | 0.066 | 0.342 | 0.062 | 0.347 |
| *g__Mitochondria* | 0.106 | 0.068 | 0.077 | 0.045 |  | 0 | 0 | 0 | 0 |
| *g__MM2* | 0.039 | 0.546 | 0.029 | 0.437 |  | 0.021 | 1.102 | 0 | 2.423 |
| *g__MND1* | 0 | 0 | 0 | 0 |  | 0 | 0.026 | 0 | 0.087 |
| *g__Mucilaginibacter* | 1.076 | 0.280 | 3.468 | 0.237 |  | 1.776 | 0.007 | 3.106 | 0 |
| *g__Mycobacterium* | 0.219 | 0.225 | 0.138 | 0.065 |  | 1.518 | 0.637 | 1.313 | 0.608 |
| *g__Myxococcus* | 0 | 0.026 | 0 | 0 |  | 0 | 0.096 | 0 | 0.070 |
| *g__Nakamurella* | 0.057 | 0 | 0.066 | 0 |  | 0.046 | 0.030 | 0.028 | 0.040 |
| *g__Nannocystis* | 0 | 0.063 | 0 | 0 |  | 0 | 0 | 0 | 0 |
| *g__Niastella* | 0.072 | 0 | 0.194 | 0 |  | 0.208 | 0 | 0.431 | 0 |
| *g__Nitrosospira* | 0.011 | 0.016 | 0 | 0 |  | 0.230 | 0.174 | 0.108 | 0.191 |
| *g__Nitrospira* | 0 | 0 | 0 | 0 |  | 0.050 | 0 | 0.040 | 0 |
| *g__Nocardioides* | 0.458 | 1.588 | 0.401 | 2.662 |  | 0.235 | 0.067 | 0.259 | 0.048 |
| *g__Nordella* | 0 | 0 | 0 | 0.006 |  | 0 | 0.090 | 0 | 0.125 |
| *g__Noviherbaspirillum* | 0.111 | 0 | 0.247 | 0 |  | 0.428 | 0.118 | 0.250 | 0.348 |
| *g__Novosphingobium* | 4.916 | 4.726 | 8.656 | 4.537 |  | 0.527 | 0.066 | 1.082 | 0.085 |
| *g__Obscuribacteraceae* | 0.006 | 0 | 0.006 | 0 |  | 0.139 | 0 | 0.377 | 0 |
| *g__Occallatibacter* | 0 | 0 | 0.098 | 0 |  | 0.201 | 0 | 0.363 | 0 |
| *g__Ohtaekwangia* | 0.042 | 0.730 | 0.010 | 0.377 |  | 0.072 | 0.663 | 0 | 1.079 |
| *g__Oligoflexus* | 0 | 0 | 0 | 0 |  | 0 | 0.020 | 0 | 0.034 |
| *g__Opitutus* | 0.032 | 0.254 | 0.006 | 0.046 |  | 0 | 0.005 | 0.003 | 0.004 |
| *g__Oxalophagus* | 0 | 0 | 0 | 0 |  | 0 | 0 | 0.060 | 0 |
| *g__P3OB-42* | 0.032 | 0 | 0.016 | 0.046 |  | 0.042 | 0 | 0.025 | 0.005 |
| *g__Paenarthrobacter* | 0.338 | 0.673 | 0.058 | 1.069 |  | 0 | 0.037 | 0 | 0.020 |
| *g__Paenibacillus* | 0.445 | 0.329 | 0.247 | 0.254 |  | 1.024 | 0.750 | 0.976 | 0.578 |
| *g__Paenisporosarcina* | 0 | 0.066 | 0 | 0.037 |  | 0 | 0.231 | 0 | 0.090 |
| *g__Pajaroellobacter* | 0.030 | 0 | 0 | 0 |  | 0.093 | 0 | 0.088 | 0 |
| *g__Paludibaculum* | 0 | 0 | 0 | 0 |  | 0.020 | 0.069 | 0 | 0.064 |
| *g__Pandoraea* | 0.265 | 0 | 0.058 | 0 |  | 0.542 | 0 | 0.105 | 0 |
| *g__Parafilimonas* | 0 | 0 | 0 | 0 |  | 0.070 | 0 | 0.033 | 0 |
| *g__Parasegetibacter* | 0.007 | 0.249 | 0.017 | 0.181 |  | 0 | 0.003 | 0 | 0.010 |
| *g__Paucibacter* | 0 | 0 | 0.057 | 0 |  | 0 | 0 | 0.016 | 0 |
| *g__Pedobacter* | 0.027 | 0.101 | 0 | 0.078 |  | 0.332 | 0.459 | 0.051 | 0.324 |
| *g__Pedomicrobium* | 0 | 0 | 0 | 0 |  | 0 | 0.024 | 0 | 0.027 |
| *g__Pedosphaera* | 0 | 0 | 0.009 | 0 |  | 0.127 | 0.038 | 0.374 | 0.083 |
| *g__Pedosphaeraceae* | 0.119 | 0.145 | 0.011 | 0.037 |  | 1.185 | 0.293 | 1.244 | 0.505 |
| *g__Peredibacter* | 0.049 | 0.088 | 0 | 0.151 |  | 0 | 0.022 | 0 | 0.058 |
| *g__Phenylobacterium* | 0.068 | 0.117 | 0.110 | 0.173 |  | 0.053 | 0.128 | 0.167 | 0.112 |
| *g__Phycicoccus* | 0.204 | 0 | 0.067 | 0 |  | 0 | 0 | 0 | 0 |
| *g__Phyllobacterium* | 0 | 0.424 | 0 | 0.369 |  | 0 | 0.995 | 0 | 0.828 |
| *g__PLTA13* | 0 | 0 | 0 | 0 |  | 0 | 0.065 | 0 | 0.075 |
| *g__Polyangium* | 0 | 0.201 | 0 | 0.019 |  | 0 | 0 | 0 | 0 |
| *g__Pontibacter* | 0 | 0 | 0 | 0.057 |  | 0 | 0.059 | 0 | 0.132 |
| *g__possible_genus_04* | 0.009 | 0 | 0 | 0 |  | 0.163 | 0 | 0.007 | 0 |
| *g__Promicromonospora* | 0 | 0.169 | 0 | 0.419 |  | 0 | 0.030 | 0 | 0.030 |
| *g__Pseudarthrobacter* | 0.213 | 0 | 0.040 | 0 |  | 0 | 0 | 0 | 0 |
| *g__Pseudolabrys* | 0 | 0 | 0 | 0 |  | 0.007 | 0 | 0.026 | 0 |
| *g__Pseudomonas* | 10.239 | 8.585 | 4.260 | 19.858 |  | 0.850 | 2.598 | 0.182 | 5.278 |
| *g__Pseudonocardia* | 0.021 | 0 | 0.192 | 0.077 |  | 0.295 | 0.076 | 0.274 | 0.088 |
| *g__Pseudorhodoferax* | 0 | 0.060 | 0 | 0.012 |  | 0 | 0 | 0 | 0 |
| *g__Pseudorhodoplanes* | 0 | 0 | 0 | 0 |  | 0 | 0.041 | 0 | 0.047 |
| *g__Pseudoxanthomonas* | 0.163 | 2.395 | 0.077 | 2.848 |  | 0 | 0.568 | 0 | 0.516 |
| *g__Puia* | 0.008 | 0.007 | 0.029 | 0.014 |  | 0.045 | 0.013 | 0.115 | 0.016 |
| *g__Qipengyuania* | 0.077 | 0 | 0 | 0 |  | 0 | 0 | 0 | 0 |
| *g__Rahnella1* | 0.198 | 0.032 | 0.064 | 0 |  | 0 | 0 | 0 | 0 |
| *g__Ralstonia* | 0.157 | 0.069 | 0.471 | 0.064 |  | 0.049 | 0.096 | 0.141 | 0.136 |
| *g__Ramlibacter* | 0.226 | 0.054 | 0.393 | 0 |  | 2.800 | 0.059 | 1.380 | 0.062 |
| *g__RB41* | 0.005 | 0.168 | 0 | 0.062 |  | 0.375 | 2.265 | 0.320 | 2.652 |
| *g__RCP2-54* | 0 | 0 | 0 | 0 |  | 0 | 0.027 | 0 | 0.051 |
| *g__Reyranella* | 0.013 | 0.061 | 0.018 | 0.020 |  | 0.124 | 0.491 | 0.141 | 0.369 |
| *g__Rheinheimera* | 0 | 0 | 0.006 | 0.043 |  | 0 | 0.057 | 0 | 0.046 |
| *g__Rhizobacter* | 0.054 | 0.139 | 0.011 | 0.018 |  | 0.006 | 0.018 | 0 | 0.041 |
| *g__Rhodococcus* | 0.327 | 0.304 | 0.499 | 1.006 |  | 0.020 | 0.007 | 0.011 | 0 |
| *g__Rhodomicrobium* | 0 | 0 | 0 | 0 |  | 0 | 0.014 | 0 | 0.026 |
| *g__Rhodoplanes* | 0 | 0.066 | 0 | 0 |  | 0 | 0.786 | 0.020 | 0.687 |
| *g__Roseiarcus* | 0 | 0 | 0.031 | 0 |  | 0.249 | 0 | 0.220 | 0 |
| *g__Roseimicrobium* | 0 | 0.010 | 0 | 0.041 |  | 0 | 0.005 | 0 | 0.006 |
| *g__Rubrobacter* | 0 | 0 | 0 | 0 |  | 0 | 0.040 | 0 | 0.066 |
| *g__Rugosimonospora* | 0 | 0 | 0 | 0 |  | 0.049 | 0 | 0.080 | 0 |
| *g__Rurimicrobium* | 0.033 | 0 | 0.068 | 0 |  | 0 | 0 | 0 | 0 |
| *g__S0134_terrestrial_group* | 0 | 0 | 0 | 0 |  | 0 | 0.060 | 0 | 0.097 |
| *g__Saccharimonadales* | 0.131 | 0.007 | 0.064 | 0 |  | 0.038 | 0.006 | 0.059 | 0.028 |
| *g__Sandaracinus* | 0 | 0.012 | 0 | 0.033 |  | 0 | 0.004 | 0 | 0.017 |
| *g__Sanguibacter* | 0 | 0 | 0 | 0.074 |  | 0 | 0 | 0 | 0 |
| *g__SC-I-84* | 0 | 0.008 | 0 | 0.004 |  | 0 | 0.275 | 0 | 0.231 |
| *g__Sediminibacterium* | 0.006 | 0.030 | 0.022 | 0 |  | 0.003 | 0.020 | 0.014 | 0.028 |
| *g__Segetibacter* | 0.037 | 0 | 0.016 | 0 |  | 0.149 | 0 | 0.113 | 0 |
| *g__Shimazuella* | 0 | 0 | 0.003 | 0 |  | 0.056 | 0 | 0.055 | 0 |
| *g__Shinella* | 0.213 | 0.653 | 0 | 0.727 |  | 0.008 | 0 | 0 | 0.034 |
| *g__Skermanella* | 0.150 | 0.057 | 0.063 | 0.105 |  | 0.040 | 0 | 0.004 | 0 |
| *g__SM2D12* | 0 | 0.082 | 0 | 0.029 |  | 0 | 0 | 0 | 0 |
| *g__Solibacillus* | 0 | 0.037 | 0.020 | 0.062 |  | 0 | 0.008 | 0 | 0.019 |
| *g__Solirubrobacter* | 0 | 0 | 0.016 | 0 |  | 0.015 | 0 | 0.028 | 0.031 |
| *g__Solitalea* | 0 | 0.037 | 0 | 0.028 |  | 0 | 0.150 | 0 | 0.183 |
| *g__Sphingobium* | 1.438 | 2.110 | 0.441 | 2.013 |  | 0 | 0.005 | 0 | 0.004 |
| *g__Sphingomonas* | 1.697 | 1.245 | 1.904 | 0.683 |  | 2.175 | 1.203 | 1.523 | 0.758 |
| *g__Sphingopyxis* | 0.011 | 0.121 | 0.030 | 0.097 |  | 0 | 0 | 0 | 0 |
| *g__Sporacetigenium* | 0 | 0.025 | 0 | 0.020 |  | 0 | 1.293 | 0 | 1.529 |
| *g__Sporocytophaga* | 0.015 | 0 | 0.047 | 0 |  | 0.944 | 0 | 1.453 | 0.037 |
| *g__Sporosarcina* | 0 | 0 | 0.006 | 0 |  | 0.037 | 0 | 0.052 | 0 |
| *g__Stenotrophomonas* | 0.013 | 0.114 | 0 | 0.297 |  | 0.003 | 0 | 0 | 0.010 |
| *g__Steroidobacter* | 0 | 0.088 | 0 | 0.071 |  | 0 | 0.938 | 0 | 0.833 |
| *g__Streptomyces* | 0.386 | 1.329 | 0.453 | 1.611 |  | 0.507 | 0.415 | 0.382 | 0.482 |
| *g__Streptosporangium* | 0 | 0.015 | 0 | 0 |  | 0.014 | 0.106 | 0.045 | 0.071 |
| *g__Subgroup_10* | 0 | 0.004 | 0 | 0.004 |  | 0 | 0.095 | 0 | 0.068 |
| *g__Subgroup_2* | 0.018 | 0 | 0 | 0 |  | 0.051 | 0 | 0.078 | 0 |
| *g__Subgroup_5* | 0.005 | 0 | 0 | 0.006 |  | 0.019 | 0.247 | 0.006 | 0.325 |
| *g__Subgroup_7* | 0 | 0 | 0 | 0 |  | 0 | 0.101 | 0 | 0.071 |
| *g__Tahibacter* | 0 | 0.047 | 0 | 0 |  | 0 | 0.028 | 0 | 0 |
| *g__Taibaiella* | 0.103 | 0 | 0.058 | 0 |  | 0 | 0 | 0 | 0 |
| *g__Tardiphaga* | 0.019 | 0.028 | 0 | 0.020 |  | 0 | 0 | 0.012 | 0 |
| *g__Terrabacter* | 0.463 | 0 | 0.317 | 0.016 |  | 0.023 | 0 | 0.021 | 0 |
| *g__Terrimonas* | 0 | 0.135 | 0 | 0.041 |  | 0 | 0.186 | 0 | 0.239 |
| *g__Thermomonas* | 0.083 | 0.060 | 0.264 | 0.066 |  | 0.004 | 0.074 | 0 | 0.060 |
| *g__TK10* | 0 | 0 | 0 | 0 |  | 0 | 0.012 | 0 | 0.055 |
| *g__TM7a* | 0.255 | 1.209 | 0.268 | 0.841 |  | 0.052 | 0.002 | 0.046 | 0.021 |
| *g__TRA3-20* | 0 | 0 | 0.004 | 0 |  | 0.382 | 0.255 | 0.197 | 0.262 |
| *g__Tumebacillus* | 0.704 | 1.205 | 0.435 | 0.412 |  | 12.347 | 4.749 | 22.809 | 3.259 |
| *g__Uliginosibacterium* | 0.039 | 0 | 0 | 0.016 |  | 0.041 | 0 | 0.007 | 0 |
| *g__Variovorax* | 0.089 | 0.266 | 0.241 | 0.146 |  | 0.022 | 0 | 0.075 | 0.003 |
| *g__Verrucomicrobium* | 0.018 | 0.155 | 0.007 | 0.088 |  | 0 | 0.021 | 0 | 0.043 |
| *g__Vicinamibacter* | 0 | 0.015 | 0 | 0.020 |  | 0 | 0.080 | 0 | 0.050 |
| *g__Vicinamibacteraceae* | 0 | 0.053 | 0 | 0.012 |  | 0.092 | 1.376 | 0.012 | 1.342 |
| *g__Vogesella* | 0 | 0 | 0 | 0.032 |  | 0 | 0.017 | 0 | 0.439 |
| *g__WCHB1-32* | 0 | 0.002 | 0 | 0 |  | 0.012 | 0.109 | 0.018 | 0.025 |
| *g__WD2101_soil_group* | 0.016 | 0 | 0 | 0 |  | 0.030 | 0 | 0.041 | 0 |
| *g__Zoogloea* | 0.178 | 0.014 | 0.023 | 0.034 |  | 0 | 0 | 0.004 | 0 |
| *g__uncultured* | 1.227 | 1.718 | 3.008 | 1.053 |  | 7.118 | 8.933 | 6.787 | 8.698 |

Table S5. Differential abundance analysis at genus level in both rhizosphere and rhizoplane compartments of soil substrates, considering the comparisons: non-urban vs peri-urban, non-urban vs non-urban + Biochar (B), peri-urban vs peri-urban + B. Statistical significance (*p*$\leq$*0.01*) was determined using ANOVA.

| **Compartment** | **Comparison** | **Genus** | ***p-value*** | **Log2FC** |
| --- | --- | --- | --- | --- |
| **Rhizoplane** | non-urban vs peri- urban | *Methylorosula* | *0.00705* | *9.8221* |
|  |  | *Chryseolinea* | *0.002068* | *-9.5613* |
|  |  | *Solibacillus* | *0.007655* | *-8.5318* |
|  |  | *C0119* | *0.005113* | *8.4549* |
|  |  | *Domibacillus* | *0.001313* | *-8.4224* |
| **Rhizosphere** | non-urban vs peri- urban | *Sporacetigenium* | *0.000001* | *-13.5758* |
|  |  | *Candidatus_Koribacter* | *0.000004* | *13.3707* |
|  |  | *Phyllobacterium* | *0* | *-13.3143* |
|  |  | *Steroidobacter* | *0* | *-13.2125* |
|  |  | *Sporocytophaga* | *0.000613* | *13.2051* |
|  |  | *Rhodoplanes* | *0* | *-12.8874* |
|  |  | *Clostridium_sensu_stricto_9* | *0.002747* | *12.7669* |
|  |  | *Hydrogenophaga* | *0.00002* | *-12.619* |
|  |  | *Pseudoxanthomonas* | *0.002435* | *-12.5712* |
|  |  | *Pandoraea* | *0.000017* | *12.4042* |
|  |  | *Micromonospora* | *0* | *-12.3948* |
|  |  | *Dongia* | *0* | *-12.3518* |
|  |  | *bacteriap25* | *0* | *-12.1824* |
|  |  | *Acidipila* | *0* | *12.1382* |
|  |  | *Agromyces* | *0* | *-12.0895* |
|  |  | *Chryseolinea* | *0* | *-11.8595* |
|  |  | *ADurb.Bin063.1* | *0.000011* | *11.7853* |
|  |  | *LWQ8* | *0.001377* | *11.6159* |
|  |  | *Asticcacaulis* | *0.000133* | *11.5344* |
|  |  | *SC.I.84* | *0* | *-11.4106* |
|  |  | *Flavitalea* | *0* | *-11.3048* |
|  |  | *Roseiarcus* | *0* | *11.2854* |
|  |  | *Paenisporosarcina* | *0* | *-11.1473* |
|  |  | *Caenimonas* | *0.001955* | *11.0637* |
|  |  | *Niastella* | *0.002697* | *11.0259* |
|  |  | *Terrimonas* | *0.000116* | *-11.0259* |
|  |  | *JG30.KF.CM45* | *0.000194* | *-10.9945* |
|  |  | *Occallatibacter* | *0.003507* | *10.9723* |
|  |  | *Acidibacter* | *0.000095* | *-10.9368* |
|  |  | *Granulicella* | *0.000174* | *10.8151* |
|  |  | *C0119* | *0.000296* | *10.8144* |
|  |  | *Segetibacter* | *0.00009* | *10.5398* |
|  |  | *Solitalea* | *0.000002* | *-10.4886* |
|  |  | *Ferribacterium* | *0.00287* | *-10.4386* |
|  |  | *Obscuribacteraceae* | *0.000711* | *10.4382* |
|  |  | *CCD24* | *0.004375* | *-10.4165* |
|  |  | *Methylorosula* | *0.000077* | *10.3298* |
|  |  | *Fonticella* | *0.000084* | *10.2943* |
|  |  | *Actinoallomurus* | *0.000982* | *10.1202* |
|  |  | *Bacteriovorax* | *0.00021* | *9.9083* |
|  |  | *Nordella* | *0.004371* | *-9.7553* |
|  |  | *Microlunatus* | *0.000098* | *9.4957* |
|  |  | *Parafilimonas* | *0.005631* | *9.4635* |
|  |  | *MB.A2.108* | *0.001792* | *-9.3814* |
|  |  | *Shimazuella* | *0.000095* | *9.1442* |
|  |  | *Ferruginibacter* | *0.006287* | *9.0987* |
|  |  | *Nitrospira* | *0.001697* | *8.9543* |
|  |  | *Uliginosibacterium* | *0.008745* | *8.6781* |
|  |  | *Rubrobacter* | *0.005627* | *-8.6709* |
|  |  | *Luedemannella* | *0* | *-6.5305* |
|  |  | *Ramlibacter* | *0.000005* | *5.4196* |
|  |  | *Cellvibrio* | *0.000031* | *-5.207* |
|  |  | *Acinetobacter* | *0.006217* | *-5.1096* |
|  |  | *Burkholderia.Caballeronia.Paraburkholderia* | *0.001199* | *4.6338* |
|  |  | *Devosia* | *0* | *-4.5864* |
|  |  | *Edaphobacter* | *0* | *4.5458* |
|  |  | *Kapabacteriales* | *0.009077* | *4.4458* |
|  |  | *Adhaeribacter* | *0.002692* | *-4.4059* |
|  |  | *Methylotenera* | *0.000148* | *-4.3718* |
|  |  | *Vicinamibacteraceae* | *0* | *-3.8498* |
|  |  | *Inquilinus* | *0.009894* | *-3.8491* |
|  |  | *Ktedonobacter* | *0.000001* | *3.7953* |
|  |  | *Ohtaekwangia* | *0.000003* | *-3.3038* |
|  |  | *Gemmatimonas* | *0.000001* | *3.2315* |
|  |  | *Chitinophaga* | *0.00036* | *3.1365* |
|  |  | *Kribbella* | *0* | *-3.0138* |
|  |  | *Streptosporangium* | *0.000437* | *-2.89* |
|  |  | *X67.14* | *0.000132* | *-2.4246* |
|  |  | *Pseudonocardia* | *0.000394* | *2.3032* |
|  |  | *Microvirga* | *0.000279* | *-2.293* |
|  |  | *Pedosphaeraceae* | *0.000492* | *2.0375* |
|  |  | *Reyranella* | *0.000021* | *-1.9622* |
|  |  | *Nocardioides* | *0.004746* | *1.9558* |
|  |  | *Candidatus_Xiphinematobacter* | *0.000001* | *-1.7273* |
|  |  | *Noviherbaspirillum* | *0.006356* | *1.6438* |
|  |  | *Pseudomonas* | *0.007218* | *-1.6418* |
|  |  | *Ellin517* | *0.001739* | *1.5831* |
|  |  | *Chthoniobacter* | *0.002907* | *-1.3133* |
|  |  | *Mycobacterium* | *0.000992* | *1.2822* |
|  |  | *Sphingomonas* | *0.001021* | *1.022* |
|  |  | *Candidatus_Udaeobacter* | *0.000027* | *0.8906* |
|  |  | *Bryobacter* | *0.005587* | *0.6704* |
|  | non-urban vs non - urban + B | *A21b* | *0.005* | *1.31* |
|  |  | *ADurb.Bin063.1* | *0.006* | *0.91* |
|  |  | *Inquilinus* | *0.008* | *3.1* |
|  |  | *KD4.96* | *0.008* | *-1.67* |
|  |  | *LWQ8* | *0.001* | *1.45* |
|  |  | *Microlunatus* | *0.006* | *-1.08* |
|  |  | *Obscuribacteraceae* | *0.006* | *1.44* |
|  |  | *Pandoraea* | *0.002* | *-2.36* |
|  |  | *Pedosphaera* | *0.006* | *1.55* |
|  |  | *Puia* | *0.001* | *1.36* |
|  | peri-urban vs peri-urban + B | *BIrii41* | *0.001* | *-2.36* |
|  |  | *Curvibacter* | *0.005* | *-1.53* |
|  |  | *Gemmatimonas* | *0.006* | *-1.55* |
|  |  | *MND1* | *0.004* | *-2.5* |
|  |  | *Paenisporosarcina* | *0.001* | *1.28* |

Table S6. Calculated networks features in the two soil typologies (non-urban and peri-urban), compartments (RP: rhizoplane, RS: rhizosphere) and condition (with (B) and without biochar).

|  | Non-urban | | | | Peri-urban | | | |
| --- | --- | --- | --- | --- | --- | --- | --- | --- |
|  | RP | RP + B | RS | RS + B | RP | RP + B | RS | RS +B |
| Average path length | 1.7 | 1.89 | 1.7 | 1.78 | 1.79 | 1.84 | 1.85 | 1.85 |
| Modularity | 0.39 | 0.52 | 0.4 | 0.44 | 0.41 | 0.47 | 0.5 | 0.52 |
| Clustering coefficient | 0.85 | 0.84 | 0.82 | 0.83 | 0.83 | 0.84 | 0.82 | 0.83 |
|  | Random Non-urban | | | | Random Peri-urban | | | |
|  | RP | RP + B | RS | RS + B | RP | RP + B | RS | RS +B |
| Average path length | 1.55 | 1.63 | 1.61 | 1.88 | 1.60 | 1.61 | 1.80 | 1.84 |
| Modularity | 0.54 | 0.47 | 0.50 | 0.25 | 0.50 | 0.49 | 0.28 | 0.28 |
| Clustering coefficient | 0.06 | 0.07 | 0.08 | 0.13 | 0.06 | 0.07 | 0.12 | 0.11 |

Table S7. Composition and clustering of the module for each microbial network (Rhizosphere and rhizoplane with and without biochar).

| Compartment | Biochar | Module | Genus | N. of genus |
| --- | --- | --- | --- | --- |
| Rhizoplane | No | M1 | *Acidovorax Acinetobacter Aeromonas Agromyces Alsobacter AltererythrobacterArcticibacter Azoarcus   Bacillus Bdellovibrio Burkholderia.Caballeronia.ParaburkholderiaC0119 Candidatus Cellulomonas Cellvibrio Chitinimonas   Chitinophaga Chryseolinea Clostridium Comamonas Curvibacter Deinococcus Devosia Domibacillus   Dongia Duganella Dyadobacter Enterobacter Ferrovibrio FlavihumibacterFlavisolibacterFlavitalea   Fluviicola Gemmobacter Haliangium Herbaspirillum Herpetosiphon Hydrogenophaga Ideonella Inhella   Lacibacter Limnobacter Luteolibacter Lysinibacillus Mesorhizobium MethylobacillusMethylobacterium.MethylorubrumMethylorosula   MethyloversatilisMethylovorus Microvirga Mitochondria MucilaginibacterMyxococcus Niastella Nocardioides   Ohtaekwangia Opitutus PaenisporosarcinaPandoraea ParasegetibacterPedobacter PhenylobacteriumPhycicoccus   PhyllobacteriumPolyangium PseudorhodoferaxPseudoxanthomonasRalstonia Ramlibacter RB41 Reyranella   Rhodococcus Rhodoplanes Roseimicrobium Sandaracinus SediminibacteriumShinella Solibacillus Solitalea   Sphingopyxis Steroidobacter Subgroup Tahibacter Terrabacter Terrimonas Thermomonas Uliginosibacterium  VerrucomicrobiumX37.13* | 90 |
| Rhizoplane | No | M2 | *Achromobacter Acidipila ActinoallomurusActinoplanes Aetherobacter Allorhizobium.Neorhizobium.Pararhizobium.RhizobiumalphaI Aquabacterium   Asticcacaulis Azohydromonas Azospirillum Bacteroides Brevibacillus Bryobacter Caenimonas Candidatus   Caulobacter ChryseobacteriumChthoniobacter Clostridium Cohnella Cupriavidus Curtobacterium Cytophaga   Dyella Edaphobaculum Enterobacteriaceaeenv.OPS Erwinia Filimonas FimbriimonadaceaeFlavobacterium   Fonticella Gaiella Gemmatimonas Granulicella Ilumatobacter JatrophihabitansKapabacterialesKosakonia   Ktedonobacter Legionella LWQ8 Lysobacter Massilia Methylophilus Methylotenera Nakamurella   Nitrosospira NoviherbaspirillumNovosphingobiumObscuribacteraceaeP3OB.42 Paenibacillus PajaroellobacterPedosphaeraceae  possible PseudarthrobacterPseudomonas Pseudonocardia Puia Qipengyuania Rahnella1 Rurimicrobium   SaccharimonadalesSegetibacter Skermanella Sphingomonas Sporocytophaga Subgroup Taibaiella Tardiphaga   TM7a Tumebacillus UNCLASSIFIED WD2101 Zoogloea* | 77 |
| Rhizoplane | No | M3 | *Algoriphagus Arenimonas bacteriap25 Bacteriovorax Bauldia Bosea Bradyrhizobium Candidatus   Catellatospora D05.2 DactylosporangiumDechloromonas Defluviimonas Edaphobacter Ellin517   Hirschia IMCC26256 Inquilinus JG30.KF.CM45 KD4.96 KF.JG30.B3 Kribbella Lacunisphaera   Luedemannella MB.A2.108 Micromonospora MM2 Mycobacterium Nannocystis PaenarthrobacterPeredibacter   PromicromonosporaRhizobacter SC.I.84 SM2D12 Sphingobium SporacetigeniumStenotrophomonasStreptomyces   Streptosporangiumuncultured Variovorax Vicinamibacter VicinamibacteraceaeWCHB1.32 X67.14* | 47 |
| Rhizoplane | Yes | M1 | *Achromobacter Acidovorax Actinoplanes Aeromonas AltererythrobacterAquabacterium Aquicella Arcticibacter   Arenimonas Asticcacaulis Azoarcus Azohydromonas Azospira Azospirillum Bacillus Bacteriovorax   Bdellovibrio Bosea Bradyrhizobium Candidatus Cellulomonas Cellvibrio Chitinimonas   Chryseolinea Chthoniobacter Clostridium Comamonas Cupriavidus Curtobacterium Curvibacter D05.2   Dechloromonas Dechlorosoma Defluviimonas Devosia Domibacillus Dongia Enterobacter Ferribacterium   Ferrovibrio FlavisolibacterFlavitalea Fluviicola Gemmobacter GlutamicibacterHaliangium Herpetosiphon   Hydrogenophaga JG30.KF.CM45 Kribbella Lacibacter Lactobacillus Lacunisphaera Limnobacter Luteolibacter   Lysinibacillus MB.A2.108 Methylotenera Methylovorus Microvirga MM2 Opitutus P3OB.42   Paenibacillus PaenisporosarcinaParasegetibacterPedosphaeraceaePhenylobacteriumPolyangium Puia RB41   Reyranella Rheinheimera Rhizobacter Roseimicrobium Sanguibacter SC.I.84 Skermanella SM2D12   Solibacillus Solitalea SporacetigeniumSteroidobacter Subgroup Tardiphaga TM7a Tumebacillus   UliginosibacteriumUNCLASSIFIED VerrucomicrobiumVogesella X37.13 Zoogloea* | 94 |
| Rhizoplane | Yes | M2 | *Acinetobacter ActinoallomurusAdhaeribacter ADurb.Bin063.1 Agromyces Algoriphagus Alkanibacter Allorhizobium.Neorhizobium.Pararhizobium.Rhizobium  Alsobacter AnaeromyxobacterBacteroides BIyi10 Brevundimonas Burkholderia.Caballeronia.ParaburkholderiaC0119 Caenimonas   Candidatus Catellatospora Caulobacter Cytophaga Deinococcus Duganella Dyadobacter Dyella   Filimonas FimbriimonadaceaeFlavobacterium Herbaspirillum Hirschia Inhella KapabacterialesKosakonia   Luteibacter LWQ8 Lysobacter Massilia Mesorhizobium MethylobacillusMethylobacterium.MethylorubrumMethylophilus   MethyloversatilisMitochondria MucilaginibacterNakamurella Nocardioides Nordella NoviherbaspirillumNovosphingobium  ObscuribacteraceaePaenarthrobacterPandoraea Paucibacter Pedobacter Peredibacter PhyllobacteriumPontibacter   PromicromonosporaPseudarthrobacterPseudomonas Pseudonocardia PseudorhodoferaxPseudoxanthomonasRahnella1 Ralstonia   Ramlibacter SaccharimonadalesShimazuella Shinella Sphingobium Sphingomonas Sphingopyxis Stenotrophomonas  Streptomyces Subgroup Taibaiella Terrabacter Terrimonas Thermomonas TRA3.20 uncultured   Variovorax Vicinamibacter VicinamibacteraceaeX67.14* | 84 |
| Rhizoplane | Yes | M3 | *Acidipila AKIW781 Bryobacter Candidatus Chitinophaga Edaphobacter Edaphobaculum Ellin516   Ellin517 Gaiella Gemmatimonas Granulicella Ideonella JatrophihabitansLeifsonia Methylorosula   Mycobacterium Niastella OccallatibacterOhtaekwangia Pedosphaera Phycicoccus Rhodococcus Roseiarcus   Rurimicrobium SediminibacteriumSegetibacter SolirubrobacterSporocytophaga Sporosarcina* | 30 |
| Rhizoplane | Yes | M4 | *Diaphorobacter Inquilinus Micromonospora Sandaracinus* | 4 |
| Rhizosphere | Yes | M1 | *A21b Acidibacter Acidothermus Acinetobacter ActinopolymorphaAdhaeribacter Aetherobacter Agromyces   alphaI Aquisphaera Asticcacaulis Bacillus bacteriap25 BIyi10 Bradyrhizobium Bryobacter   Burkholderia.Caballeronia.ParaburkholderiaC0119 Candidatus Catellatospora CCD24 Cellvibrio Chitinophaga   Chryseolinea Chthoniobacter Clostridium Cupriavidus D05.2 Domibacillus Dyadobacter Enterobacter   FlavisolibacterFlavitalea Flavobacterium Fluviicola Herbaspirillum JG30.KF.CM45 KD4.96 KF.JG30.B3   KI89A LWQ8 Mesorhizobium MethylobacillusMethylorosula MethyloversatilisMicrolunatus Micromonospora   Microvirga MM2 MND1 Niastella Nitrospira NoviherbaspirillumOhtaekwangia Oxalophagus   PaenarthrobacterPaenisporosarcinaParafilimonas ParasegetibacterPedobacter Peredibacter PhyllobacteriumPontibacter   Pseudomonas Pseudonocardia PseudoxanthomonasPuia RB41 RCP2.54 Rhizobacter Roseiarcus   RugosimonosporaS0134 SaccharimonadalesSandaracinus SediminibacteriumSegetibacter Shimazuella Solitalea   Sphingomonas Sporocytophaga StenotrophomonasSteroidobacter Streptomyces Subgroup Terrimonas TK10   TRA3.20 Tumebacillus Variovorax Vicinamibacter VicinamibacteraceaeWCHB1.32* | 94 |
| Rhizosphere | Yes | M2 | *Aeromonas Allorhizobium.Neorhizobium.Pararhizobium.RhizobiumAltererythrobacterAquicella Archangium Arcticibacter Arenimonas Azoarcus   Azospira Azospirillum Bacteriovorax Bauldia Bdellovibrio BIrii41 Caenimonas Caulobacter   Cellulomonas Chitinimonas CitrifermentansClostridium Cohnella Curvibacter DactylosporangiumDechlorobacter   Dechloromonas Dechlorosoma Devosia Diaphorobacter Dongia Ellin517 env.OPS Ferribacterium   Ferrovibrio Gemmobacter Herpetosiphon Hirschia Hydrogenophaga Hyphomicrobium Ilumatobacter IMCC26256   IS.44 Kribbella Lacunisphaera Limnobacter Luedemannella Luteolibacter Lysinibacillus Lysobacter   MagnetospirillumMassilia MB.A2.108 Methylotenera Myxococcus Nocardioides Nordella Oligoflexus   Paenibacillus Paludibaculum Pedomicrobium PLTA13 PromicromonosporaPseudorhodoplanesRalstonia Ramlibacter   Reyranella Rheinheimera Rhodomicrobium Rhodoplanes Rubrobacter SC.I.84 Shinella Solibacillus   SolirubrobacterSporacetigeniumStreptosporangiumSubgroup Thermomonas TM7a UNCLASSIFIED uncultured   VerrucomicrobiumVogesella X67.14* | 83 |
| Rhizosphere | Yes | M3 | *Acidipila ActinoallomurusADurb.Bin063.1 AKIW781 Alkanibacter Alsobacter Ammoniphilus Anaeromyxobacter  AurantisolimonasBosea Brevibacillus Candidatus Clostridium Cytophaga Dyella Edaphobacter   Edaphobaculum Ellin516 FerruginibacterFilimonas FimbriimonadaceaeFonticella Gemmatimonas Granulicella   Ideonella JatrophihabitansJGI KapabacterialesKtedonobacter Labrys Lactobacillus Luteibacter   Methylobacterium.MethylorubrumMethylophilus MucilaginibacterMycobacterium Nitrosospira NovosphingobiumObscuribacteraceaeOccallatibacter  P3OB.42 PajaroellobacterPandoraea Paucibacter Pedosphaera PedosphaeraceaePhenylobacteriumpossible   Pseudolabrys Rhodococcus Skermanella Sporosarcina Subgroup Tardiphaga Terrabacter Uliginosibacterium  WD2101 Zoogloea* | 58 |
| Rhizosphere | Yes | M4 | *FlavihumibacterInhella Opitutus Roseimicrobium Sphingobium* | 5 |
| Rhizosphere | No | M1 | *Achromobacter Acidovorax Actinoplanes ActinopolymorphaAeromonas AltererythrobacterAquabacterium Archangium   Arcticibacter Arenimonas Azoarcus Bacteroides Bauldia Bdellovibrio BIyi10 Catellatospora   Caulobacter CCD24 Chitinimonas Chthoniobacter CitrifermentansCL500.29 Clostridium Cohnella   Curvibacter D05.2 Dechloromonas Dechlorosoma Domibacillus Duganella Dyadobacter Ellin517   Ferribacterium Ferrovibrio FlavihumibacterFlavitalea Fluviicola Gaiella Haliangium Herpetosiphon   Hirschia Hyphomicrobium Ilumatobacter IMCC26256 IS.44 JG30.KF.CM45 KD4.96 KI89A   Kosakonia Lacibacter Luteolibacter Lysinibacillus Lysobacter MB.A2.108 Mesorhizobium Methylobacillus  Methylophilus MethyloversatilisMND1 Myxococcus Nocardioides Nordella Oligoflexus Paenarthrobacter  Paenibacillus Pedomicrobium Peredibacter PLTA13 Pontibacter PromicromonosporaPseudorhodoplanesPseudoxanthomonas  Puia RCP2.54 Reyranella Rheinheimera Rhizobacter Rhodomicrobium Rhodoplanes Rubrobacter   S0134 Sandaracinus SC.I.84 Solibacillus Sphingobium SporacetigeniumSteroidobacter Streptomyces   Tahibacter Thermomonas TK10 Tumebacillus uncultured VerrucomicrobiumVicinamibacter Vogesella   WCHB1.32* | 97 |
| Rhizosphere | No | M2 | *A21b Acidibacter Acidipila Acinetobacter ActinoallomurusAdhaeribacter ADurb.Bin063.1 Aetherobacter   Agromyces AKIW781 Alkanibacter Ammoniphilus Asticcacaulis bacteriap25 Bacteriovorax Bradyrhizobium   Bryobacter Burkholderia.Caballeronia.ParaburkholderiaC0119 Candidatus Cellvibrio Chitinophaga Chryseolinea   Cytophaga DactylosporangiumDevosia Dongia Dyella Edaphobacter Edaphobaculum Ellin516   Enterobacter env.OPS FerruginibacterFonticella Gemmatimonas Granulicella Herbaspirillum Hydrogenophaga   Ideonella KF.JG30.B3 Kribbella Lactobacillus Limnobacter Luedemannella LWQ8 Methylotenera   Micromonospora Microvirga MM2 MucilaginibacterMycobacterium Niastella Nitrospira Novosphingobium  ObscuribacteraceaeOccallatibacterOhtaekwangia PaenisporosarcinaPajaroellobacterPaludibaculum Pandoraea Pedobacter   Pedosphaera PhyllobacteriumPseudolabrys Pseudomonas Pseudonocardia RB41 Rhodococcus Roseiarcus   RugosimonosporaSegetibacter Shimazuella Skermanella Solitalea Sphingomonas Sporocytophaga Streptosporangium  Subgroup Terrabacter Terrimonas TM7a UNCLASSIFIED Variovorax VicinamibacteraceaeX67.14* | 88 |
| Rhizosphere | No | M3 | *alphaI AnaeromyxobacterAquisphaera AurantisolimonasAzohydromonas Bosea Brevibacillus Caenimonas   Cellulomonas Clostridium FimbriimonadaceaeFlavisolibacterInquilinus JGI KapabacterialesKtedonobacter   Labrys Legionella Luteibacter Lutispora MagnetospirillumMassilia Methylobacterium.MethylorubrumMethylorosula   Microlunatus Nitrosospira NoviherbaspirillumParafilimonas Pedosphaeraceaepossible Ramlibacter Saccharimonadales  SediminibacteriumShinella SolirubrobacterSporosarcina StenotrophomonasTRA3.20 UliginosibacteriumWD2101* | 40 |
| Rhizosphere | No | M4 | *Acidothermus BIrii41 P3OB.42* | 3 |

Table S8. Significant correlations found between the roots/soils parameters, enzymatic activities and the microbial network modules in the different compartment (Rhizoplane and rhizosphere) and conditions (presence or absence of biochar).

| **Compartment** | **Biochar** | **Module** | **Trait** | **Correlation** | **Pvalue** | **Trait_Type** |
| --- | --- | --- | --- | --- | --- | --- |
| Rhizoplane | Yes | M2 | NN | -0.9428571 | 0.00480466 | Soil_Chemical |
| Rhizoplane | Yes | M3 | pH | 0.98561076 | 0.00030909 | Soil_Chemical |
| Rhizoplane | Yes | M3 | MaxD | 0.95588235 | 0.00287662 | Root |
| Rhizoplane | Yes | M3 | ß-Glu | -0.9411764 | 0.00508854 | Enzyme |
| Rhizosphere | No | M3 | TOC | -0.7761182 | 0.00015286 | Soil_Chemical |
| Rhizosphere | No | M3 | SA | 0.76181538 | 0.00023886 | Root |
| Rhizosphere | No | M3 | SRL | -0.7179248 | 0.00079336 | Root |
| Rhizosphere | No | M3 | K | -0.7179248 | 0.00079336 | Soil_Chemical |
| Rhizosphere | No | M3 | Ca | -0.7179248 | 0.00079336 | Soil_Heavy_Metals |
| Rhizosphere | No | M3 | Co | -0.7179248 | 0.00079336 | Soil_Heavy_Metals |
| Rhizosphere | No | M3 | Pb | -0.7179248 | 0.00079336 | Soil_Heavy_Metals |
| Rhizosphere | No | M3 | Ni | -0.7179248 | 0.00079336 | Soil_Heavy_Metals |
| Rhizosphere | No | M3 | Zn | -0.7179248 | 0.00079336 | Soil_Heavy_Metals |
| Rhizosphere | No | M3 | Na | -0.7125019 | 0.00090647 | Soil_Chemical |
| Rhizosphere | No | M3 | NH3.NH4 | 0.7116547 | 0.00092529 | Soil_Chemical |
| Rhizosphere | No | M3 | As | -0.7095389 | 0.00097374 | Soil_Heavy_Metals |
| Rhizosphere | No | M3 | Cr | -0.7095389 | 0.00097374 | Soil_Heavy_Metals |
| Rhizosphere | No | M3 | Cu | -0.6991145 | 0.0012444 | Soil_Heavy_Metals |
| Rhizosphere | No | M3 | Mn | -0.6991145 | 0.0012444 | Soil_Heavy_Metals |
| Rhizosphere | No | M3 | TN | -0.6803043 | 0.00189059 | Soil_Chemical |
| Rhizosphere | No | M3 | P | -0.6740342 | 0.00215945 | Root |
| Rhizosphere | No | M3 | MaxD | -0.6584281 | 0.00296804 | Root |
| Rhizosphere | No | M3 | pH | -0.6552239 | 0.00316147 | Soil_Chemical |
| Rhizosphere | No | M3 | MD | -0.6360352 | 0.00454823 | Root |
| Rhizosphere | No | M3 | TRL | 0.61133333 | 0.00702567 | Root |
| Rhizosphere | No | M3 | NetA | 0.61133333 | 0.00702567 | Root |
| Rhizosphere | No | M3 | Phos | 0.78566060 | 0.000111458 | Enzyme |
| Rhizosphere | No | M4 | ß-Glu | 0.68030426 | 0.001890592 | Enzyme |
| Rhizosphere | No | M4 | Phos | -0.69341705 | 0.001416970 | Enzyme |
| Rhizosphere | Yes | M2 | As | 0.82946103 | 2.08E-05 | Soil_Heavy_Metals |
| Rhizosphere | Yes | M2 | Cr | 0.82946103 | 2.08E-05 | Soil_Heavy_Metals |
| Rhizosphere | Yes | M2 | MD | 0.81613635 | 3.62E-05 | Root |
| Rhizosphere | Yes | M2 | Na | 0.81197606 | 4.27E-05 | Soil_Chemical |
| Rhizosphere | Yes | M2 | TN | 0.81110711 | 4.42E-05 | Soil_Chemical |
| Rhizosphere | Yes | M2 | K | 0.80570598 | 5.44E-05 | Soil_Chemical |
| Rhizosphere | Yes | M2 | Zn | 0.80156467 | 6.35E-05 | Soil_Heavy_Metals |
| Rhizosphere | Yes | M2 | Ca | 0.78689572 | 0.00010687 | Soil_Heavy_Metals |
| Rhizosphere | Yes | M2 | Cu | 0.78689572 | 0.00010687 | Soil_Heavy_Metals |
| Rhizosphere | Yes | M2 | Mn | 0.78689572 | 0.00010687 | Soil_Heavy_Metals |
| Rhizosphere | Yes | M2 | NetA | 0.77435555 | 0.00016178 | Root |
| Rhizosphere | Yes | M2 | Ni | 0.77435555 | 0.00016178 | Soil_Heavy_Metals |
| Rhizosphere | Yes | M2 | V | 0.76808546 | 0.00019716 | Root |
| Rhizosphere | Yes | M2 | TOC | 0.76518925 | 0.00021558 | Soil_Chemical |
| Rhizosphere | Yes | M2 | NH3.NH4 | -0.7555453 | 0.00028776 | Soil_Chemical |
| Rhizosphere | Yes | M2 | Co | 0.75554529 | 0.00028776 | Soil_Heavy_Metals |
| Rhizosphere | Yes | M2 | Pb | 0.75554529 | 0.00028776 | Soil_Heavy_Metals |
| Rhizosphere | Yes | M2 | SRL | -0.7492752 | 0.00034483 | Root |
| Rhizosphere | Yes | M2 | SA | 0.74927521 | 0.00034483 | Root |
| Rhizosphere | Yes | M2 | P | 0.74927521 | 0.00034483 | Root |
| Rhizosphere | Yes | M2 | MaxD | 0.74431005 | 0.00039651 | Root |
| Rhizosphere | Yes | M2 | NN | -0.736735 | 0.00048782 | Soil_Chemical |
| Rhizosphere | Yes | M2 | pH | 0.71792478 | 0.00079336 | Soil_Chemical |
| Rhizosphere | Yes | M2 | TP | 0.66478975 | 0.00261276 | Soil_Chemical |
| Rhizosphere | Yes | M2 | ß-Glu | 0.81824614 | 0.002792171 | Enzyme |
| Rhizosphere | Yes | M2 | Phos | -0.66149401 | 3.32E-05 | Enzyme |
| Rhizosphere | Yes | M3 | As | -0.829461 | 2.08E-05 | Soil_Heavy_Metals |
| Rhizosphere | Yes | M3 | Cr | -0.829461 | 2.08E-05 | Soil_Heavy_Metals |
| Rhizosphere | Yes | M3 | K | -0.8119761 | 4.27E-05 | Soil_Chemical |
| Rhizosphere | Yes | M3 | Zn | -0.8111071 | 4.42E-05 | Soil_Heavy_Metals |
| Rhizosphere | Yes | M3 | Na | -0.805706 | 5.44E-05 | Soil_Chemical |
| Rhizosphere | Yes | M3 | MD | -0.8028117 | 6.06E-05 | Root |
| Rhizosphere | Yes | M3 | TN | -0.8015647 | 6.35E-05 | Soil_Chemical |
| Rhizosphere | Yes | M3 | NetA | -0.7868957 | 0.00010687 | Root |
| Rhizosphere | Yes | M3 | Ni | -0.7868957 | 0.00010687 | Soil_Heavy_Metals |
| Rhizosphere | Yes | M3 | Ca | -0.7743555 | 0.00016178 | Soil_Heavy_Metals |
| Rhizosphere | Yes | M3 | Cu | -0.7743555 | 0.00016178 | Soil_Heavy_Metals |
| Rhizosphere | Yes | M3 | Mn | -0.7743555 | 0.00016178 | Soil_Heavy_Metals |
| Rhizosphere | Yes | M3 | TOC | -0.7651893 | 0.00021558 | Soil_Chemical |
| Rhizosphere | Yes | M3 | V | -0.7555453 | 0.00028776 | Root |
| Rhizosphere | Yes | M3 | P | -0.7555453 | 0.00028776 | Root |
| Rhizosphere | Yes | M3 | NH3.NH4 | 0.74927521 | 0.00034483 | Soil_Chemical |
| Rhizosphere | Yes | M3 | Co | -0.7492752 | 0.00034483 | Soil_Heavy_Metals |
| Rhizosphere | Yes | M3 | Pb | -0.7492752 | 0.00034483 | Soil_Heavy_Metals |
| Rhizosphere | Yes | M3 | SRL | 0.73673504 | 0.00048782 | Root |
| Rhizosphere | Yes | M3 | SA | -0.736735 | 0.00048782 | Root |
| Rhizosphere | Yes | M3 | MaxD | -0.7347676 | 0.00051422 | Root |
| Rhizosphere | Yes | M3 | NN | 0.73046495 | 0.00057616 | Soil_Chemical |
| Rhizosphere | Yes | M3 | pH | -0.7116547 | 0.00092529 | Soil_Chemical |
| Rhizosphere | Yes | M3 | TP | -0.6488857 | 0.00357459 | Soil_Chemical |
| Rhizosphere | Yes | M3 | Phos | 0.73046495 | 0.00057615 | Enzyme |
